# CRISPR/Cas9-generated mutations in a sugar transporter gene reduce cassava susceptibility to bacterial blight

**DOI:** 10.1093/plphys/kiae243

**Published:** 2024-05-03

**Authors:** Kiona Elliott, Kira M Veley, Greg Jensen, Kerrigan B Gilbert, Joanna Norton, Lukas Kambic, Marisa Yoder, Alex Weil, Sharon Motomura-Wages, Rebecca S Bart

**Affiliations:** Donald Danforth Plant Science Center, Saint Louis, MO 63132, USA; Division of Biological and Biomedical Sciences, Washington University in Saint Louis, St. Louis, MO 63110, USA; Donald Danforth Plant Science Center, Saint Louis, MO 63132, USA; Donald Danforth Plant Science Center, Saint Louis, MO 63132, USA; Donald Danforth Plant Science Center, Saint Louis, MO 63132, USA; College of Tropical Agriculture & Human Resources, University of Hawaii at Manoa, Hilo, HI 96720, USA; College of Tropical Agriculture & Human Resources, University of Hawaii at Manoa, Hilo, HI 96720, USA; Donald Danforth Plant Science Center, Saint Louis, MO 63132, USA; Donald Danforth Plant Science Center, Saint Louis, MO 63132, USA; College of Tropical Agriculture & Human Resources, University of Hawaii at Manoa, Hilo, HI 96720, USA; Donald Danforth Plant Science Center, Saint Louis, MO 63132, USA

## Abstract

Bacteria from the genus *Xanthomonas* are prolific phytopathogens that elicit disease in over 400 plant species. Xanthomonads carry a repertoire of specialized proteins called transcription activator-like (TAL) effectors that promote disease and pathogen virulence by inducing the expression of host susceptibility (S) genes. *Xanthomonas phaseoli* pv. *manihotis* (*Xpm*) causes bacterial blight on the staple food crop cassava (*Manihot esculenta* Crantz). The *Xpm* effector TAL20 induces ectopic expression of the S gene *Manihot esculenta Sugars Will Eventually be Exported Transporter 10a* (*MeSWEET10a*), which encodes a sugar transporter that contributes to cassava bacterial blight (CBB) susceptibility. We used CRISPR/Cas9 to generate multiple cassava lines with edits to the *MeSWEET10a* TAL20 effector binding site and/or coding sequence. In several of the regenerated lines, *MeSWEET10a* expression was no longer induced by *Xpm*, and in these cases, we observed reduced CBB disease symptoms post *Xpm* infection. Because *MeSWEET10a* is expressed in cassava flowers, we further characterized the reproductive capability of the *MeSWEET10a* promoter and coding sequence mutants. Lines were crossed to themselves and to wild-type plants. The results indicated that expression of *MeSWEET10a* in female, but not male, flowers is critical to produce viable F1 seed. In the case of promoter mutations that left the coding sequence intact, viable F1 progeny were recovered. Taken together, these results demonstrate that blocking *MeSWEET10a* induction is a viable strategy for decreasing cassava susceptibility to CBB and that ideal lines will contain promoter mutations that block TAL effector binding while leaving endogenous expression of *MeSWEET10a* unaltered.

## Teaser

Introducing mutations into a cassava sugar transporter gene via genome editing results in decreased susceptibility to bacterial blight.

## Introduction

Cassava (*Manihot esculenta* Crantz) is a starchy root crop that serves as a carbohydrate source and food security crop for nearly 800 million people globally (Alves 2002; Morgan and Choct 2016). Cassava is tolerant to abiotic stressors and is often grown without costly inputs like fertilizer (EL-Sharkawy 2003). This crop is especially important for smallholder farmers in sub-Saharan Africa who grow cassava as a sustenance crop and sell it for revenue when yields allow (Hillocks et al. 2002; Taylor et al. 2004). A leading biotic factor threatening cassava production is cassava bacterial blight (CBB; Lozano 1986). CBB disease symptoms include water-soaked leaf lesions, chlorosis, defoliation, and stem browning (Lozano 1986). CBB is present in all cassava-growing regions and can result in total crop loss including the stem used to plant a subsequent crop through clonal propagation (Lozano et al. 1980; López and Bernal 2012).

The causal agent of CBB is a gram-negative phytopathogen in the genus *Xanthomonas*. Xanthomonads elicit disease in over 400 plant species including economically important crops such as rice (*Oryza sativa*), cotton (*Gossypium* sp.), sorghum (*Sorghum bicolor*), and citrus (*Citrus* sp.; Leyns et al. 1984; Mhedbi-Hajri et al. 2013; Jacques et al. 2016). The *Xanthomonas* specific to cassava was recently reclassified as *Xanthomonas phaseoli* pv*. manihotis* (*Xpm*; Constantin et al. 2016) and was formerly known as *Xanthomonas axonopodis* pv. *manihotis* (*Xam*). *Xpm* is dispersed from plant to plant through rain, wind, or by the propagation of already infected stem cuttings. From the leaf surface, *Xpm* can enter the leaf through open stomata or wounds (Kandel et al. 2017). In planta, *Xanthomonas* colonizes the surface of mesophyll cells and some xanthomonads, including *Xpm*, can systemically spread throughout the plant vasculature (Ryan et al. 2011; An et al. 2020).

*Xpm* induces effector-triggered susceptibility (ETS) using an arsenal of effector proteins released into the plant through a needle-like projection that penetrates the host cell wall called the type III secretion system (T3SS; Bart et al. 2012; Abrusci et al. 2014). T3SS effectors manipulate the host to help the pathogen overcome plant defenses and promote disease (Hogenhout et al. 2009). Bacteria in the *Xanthomonas* and *Ralstonia* genera have specialized transcription activator-like (TAL) effectors that induce expression of host susceptibility (S) genes to enhance pathogenesis (Eckardt 2002; van Schie and Takken 2014). TAL effectors structurally resemble eukaryotic transcription factors with an activation domain, nuclear localization signal, and a DNA-binding domain consisting of tandem amino acid repeats (Schornack et al. 2013). The DNA-binding domain directs the TAL effector to predictable DNA sequences, called effector-binding elements (EBEs; Boch et al. 2009; Moscou and Bogdanove 2009; Boch and Bonas 2010; Cernadas et al. 2014). In many cases, TAL effector binding causes upregulation of downstream susceptibility genes.

*Xpm* strains typically carry between 1 and 5 TAL effectors, and the model *Xpm* strain used in this study, Xpm668 (formerly known as Xam668), has 5 TAL effectors: TAL13, TAL14, TAL15, TAL20, and TAL22 (Cohn et al. 2014). *Xpm* TAL20 mutants (Xpm668ΔTal20) show reduced virulence with the most obvious phenotype being a reduction in the water-soaked lesions that are typical of this disease (Cohn et al. 2014). Susceptibility genes targeted by TAL20 were identified using a transcriptomic analysis of cassava infected with Xpm668 with and without TAL20, coupled with the use of TAL EBE prediction software (Cohn et al. 2014). A member of the *Sugars Will Eventually be Exported Transporter* (*SWEET*) gene family, *MeSWEET10a* (gene ID: Manes.06G123400), was identified as the S gene target for TAL20. *MeSWEET10a* was confirmed as a genuine TAL effector target using electromobility shift assays to show direct interaction of TAL20 with the *MeSWEET10a* EBE sequence. Additionally, designed TAL effectors targeting distinct places within the *MeSWEET10a* promoter were able to complement the TAL20 *Xpm* mutant (Cohn et al. 2014). TAL20 binding to the *MeSWEET10a* EBE induces ectopic gene expression in the leaf. Because *MeSWEET10a* is a predicted membrane-bound sugar transporter, ectopic expression in the leaf is presumed to result in sugar transport into the apoplast where *Xpm* proliferates (Cohn et al. 2014). SWEET genes have been studied as pathogen virulence factors in other pathosystems (Chen et al. 2010; Chen 2014). Furthermore, SWEET genes are established TAL effector targets in several plant species including rice, pepper, and cotton (Antony et al. 2010; Hu et al. 2014; Cox et al. 2017; Phillips et al. 2017), and in several cases, preventing TAL effector binding reduced plant susceptibility to disease (Oliva et al. 2019; Gupta et al. 2021; Veley et al. 2023). Additional classes of susceptibility genes have been described from various systems, for example *Cs LATERAL ORGAN BOUNDARIES 1* (*LOB1*), which is a TAL-induced target of citrus canker in sweet orange (Huang et al. 2022).

In this study, we used a dual gRNA CRISPR/Cas9 strategy to generate MeSWEET10a mutant lines with edits to the TAL20 EBE and/or gene coding sequence. We characterized the disease phenotypes of *Xpm*-infected plants and demonstrated that MeSWEET10a mutants exhibit reduced CBB symptoms. Additionally, while *MeSWEET10a* is not normally expressed in cassava leaves, prior work showed there is endogenous expression in flowers (Perera et al. 2012; Veley et al. 2021). In rice, knocking out the SWEET gene, *OsSWEET15*, led to reduced rice fertility (Hu et al. 2023). Therefore, we investigated the impact of editing *MeSWEET10a* on cassava flower development and reproductive function. We found that *MeSWEET10a* mutant cassava plants developed flowers morphologically similar to wild-type plants based on macro imaging. Lines in which the coding sequence of MeSWEET10a was disrupted were able to produce viable F1 progeny when used as the male, but not female, in crosses, suggesting that MeSWEET10a expression in female flowers may be essential. In contrast, lines with MeSWEET10a promoter mutations, which left the coding sequence intact, did produce viable F1 offspring when used as either the male or female in crosses.

## Results

**Figure 1.**
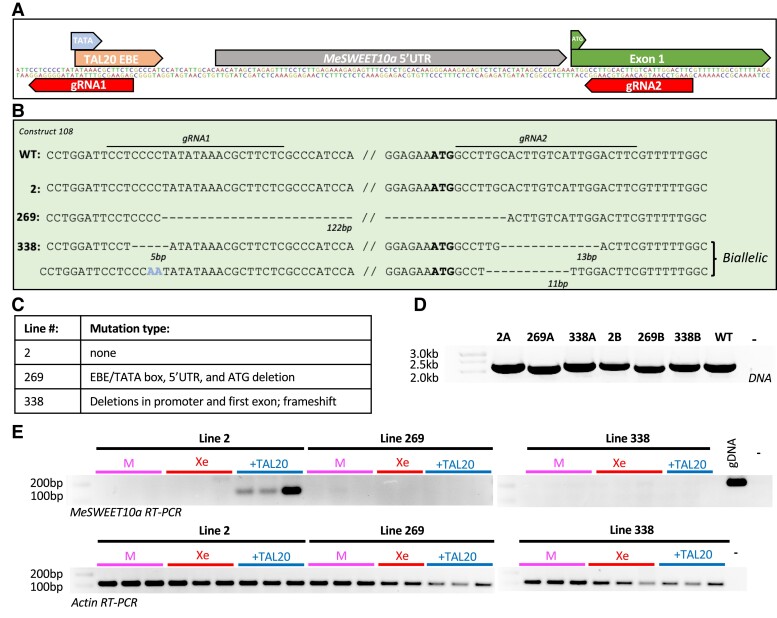
First-generation *MeSWEET10a* mutant lines lack TAL20-mediated induction. **A)** Graphic depicting the *MeSWEET10a* region of interest including the TAL20 EBE, TATA box, translation start site (ATG), and gRNA target sites for construct 108. **B)** Genotyping of *MeSWEET10a* mutant lines recovered from construct 108 based on Sanger sequencing. Text indicates sequences at the region of interest for wild-type plants and mutant lines #2, 269, and 338. The location of each gRNA target site and the corresponding gRNA number are noted. Deletions are indicated by “-” and the number of deleted base pairs (bp) is indicated below each deletion. Insertion or base substitution events are noted. The “biallelic” text to the right of the sequence distinguishes lines that are heterozygous for mutant alleles. **C)** Table with description of mutation and location type for each mutant line. **D)** PCR products generated by primers targeting the *MeSWEET10a* region in gDNA from WT and lines 2, 269, and 338. A and B denote different individuals from each line. **E)** RT-PCR of wild-type cassava and *MeSWEET10a* mutant lines infiltrated with mock, *Xe* alone, and Xe + TAL20 treatments. Top gel shows results of RT-PCR with primers amplifying *MeSWEET10a* with an expected product size of 123 bp. The bottom gel shows results of RT-PCR with primers amplifying the housekeeping gene, *Actin*, as a control for sample loading with an expected product size of 125 bp. DNA from WT419 leaf tissue is included as a positive control and “-” denotes a negative water control. M (mock); Xe; +TAL20 (Xe + TAL20).

We hypothesized that editing *MeSWEET10a* would reduce cassava susceptibility to *Xpm*. To test this hypothesis, we designed a single CRISPR/Cas9 construct (construct 108) with 2 guide RNAs (gRNAs), gRNA1 and gRNA2, which target the TAL20 EBE site and the translation start site (start codon: ATG), respectively. Additionally, while previous reports demonstrate that there is low efficiency of HDR in plants (Britt and May 2003; Puchta 2005), we optimistically included a repair template with homology arms that flank the EBE to allow for potential CRISPR-mediated homology-directed repair (HDR). The repair template was designed to replace the EBE with a sequence that TAL20 would not bind while maintaining the annotated TATA box (Fig. 1A). *Agrobacterium-*mediated transformation was carried out in friable embryogenic callus (FEC) from the farmer-preferred cultivar of cassava, TME419, also referred to as WT419 (Chauhan et al. 2015). In total, 30 transgenic lines were recovered. The *MeSWEET10a* region of interest was amplified from each recovered transgenic line. Restriction digest was used to identify lines with potential EBE repair template integration and larger INDELs. If the EBE repair template was integrated, we expected it to abolish an HaeIII restriction enzyme site at the gRNA2 repair template site (Supplementary Fig. S1, A and B). Based on this analysis, transgenic lines with integration of the repair template were not recovered. However, several lines exhibited digest patterns different from WT419 (for example lines #269 and #338; Supplementary Fig. S1C). These lines were genotyped using Sanger sequencing along with line #2, which showed a wild-type-like digest pattern (Supplementary Fig. S2A). Genotyping revealed that line #2 is a WT-like transgenic (Fig. 1, B and C). Line #269 has a large 122 bp deletion including the TATA box, TAL20 EBE, and the *MeSWEET10a* start codon consistent with the observed smaller PCR product (Fig. 1D). Line #338 is a biallelic mutant with 1 allele containing a 5 bp deletion upstream of the TATA box/TAL20 EBE and a 13 bp deletion after the start codon causing a frameshift and stop codon in exon 1. The second allele has 2 INDELS upstream of the TATA box/TAL20 EBE and a 11 bp deletion after the start codon. To confirm edit types in lines #269 and #338, both genomic DNA (gDNA) clone sequencing (Supplementary Fig. S2B) and whole genome resequencing were used (Supplementary Fig. S3, A and B). For the former, PCR was used to amplify the target region, and then the PCR product was cloned into a plasmid and transformed into *Escherichia coli.* This allowed the 2 haplotypes to be resolved independently. For the resequencing analysis, genome DNA was sequenced, and reads were mapped to the cassava reference genome, as described in the Materials and methods. These analyses confirmed the biallelic mutations in line #338 and the 122 bp deletion in line #269. It is possible, albeit unlikely, that line #269 is homozygous for the deletion. Alternatively, the second allele may contain a more complicated edit that was not resolved through these methods.

It was previously demonstrated that a TAL effectorless xanthomonad, *Xanthomonas euvesicatoria* (*Xe*—nonpathogenic to cassava), can deliver TAL20 to cassava cells and induce *MeSWEET10a* expression (Cohn et al. 2014). Use of the *Xe* system is advantageous in some experiments as it separates the impact of TAL20 away from the other TAL effectors present in *Xpm*. We used this system to compare *Xe*, Xe +TAL20, or mock treatments for *MeSWEET10a* induction in lines #2, #269, and #338. At 48 h post infection (HPI), samples were collected for RNA extraction and reverse transcription (RT)-PCR (Fig. 1E). In control line #2 plants infected with Xe +TAL20, RT-PCR results show a 123 bp product indicating TAL20-mediated induction of *MeSWEET10a*. In contrast, no product was present for plants infected with *Xe* alone or mock treatments. Mutant lines #269 and #338 infected with Xe +TAL20 have no RT-PCR product indicating the mutations in each line are sufficient to prevent TAL20-mediated induction of *MeSWEET10a.*

**Figure 2.**
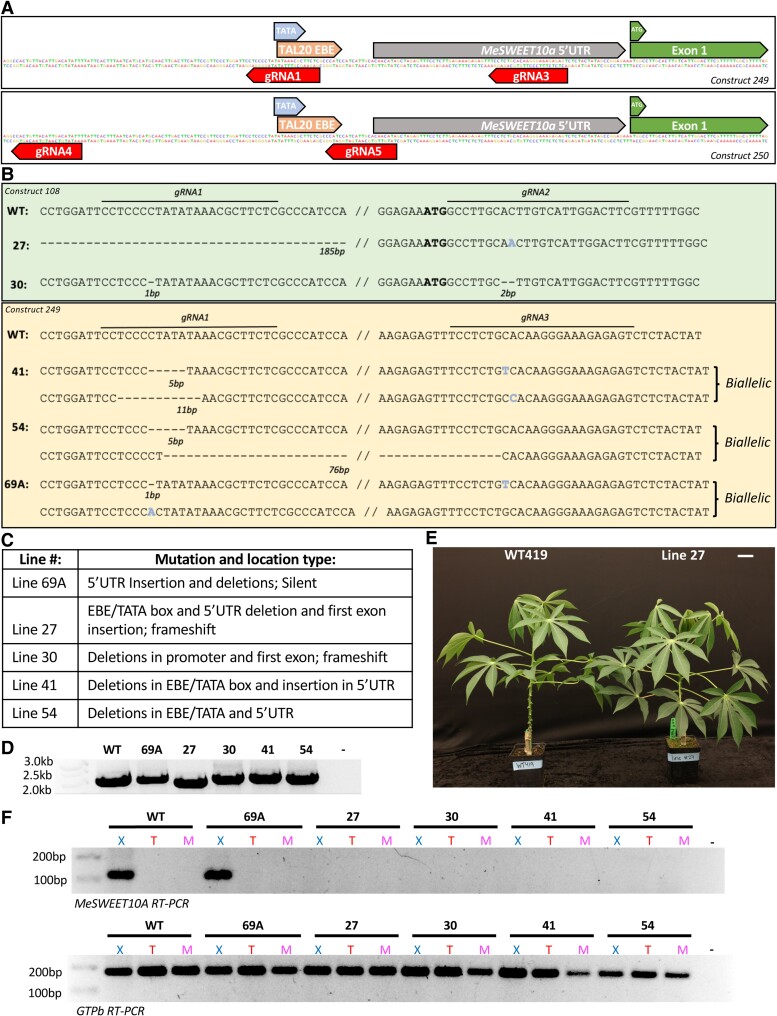
Additional *MeSWEET10a* mutant lines lack TAL20-mediated induction. **A)** Graphic depicting the *MeSWEET10a* region of interest, TAL20 EBE site, TATA box, translation start site (ATG), and gRNA target sites for constructs 249 and 250. **B)** Genotyping of *MeSWEET10a* mutant lines recovered from constructs 108 and 249 based on Sanger sequencing. Text indicates sequences at the region of interest for wild-type plants and mutant lines #27, 30, 41, 54, and 69A. The location of each gRNA target site and the corresponding gRNA number are noted. Deletions are indicated by “-” and the number of deleted base pairs (bp) is indicated below each deletion. Insertion or base substitution events are noted. The “biallelic” text to the right of the sequence distinguishes lines that are heterozygous for mutant alleles. **C)** Table with description of mutation and location type for each mutant line. **D)** PCR products generated by primers targeting the *MeSWEET10a* region in gDNA from WT and edited lines. **E)** Representative image of wild-type cassava (left) and line 27 (right) plants grown from stake cuttings in greenhouse. Scale bar = 14 cm. **F)** RT-PCR of wild-type cassava and *MeSWEET10a* mutant lines infected with *Xpm*, Xpm△TAL20, and mock treatments. The top gel shows results of RT-PCR with primers amplifying *MeSWEET10a* with an expected product size of 123 bp. The bottom gel shows results of RT-PCR with primers amplifying the housekeeping gene GTPB as a control for sample loading with an expected product size of 184 bp. “-” denotes a negative water control. X, *Xpm*; T, Xpm△TAL20; M, mock.

Two additional CRISPR/Cas9 constructs were tested (Fig. 2A). Construct 249 contains gRNA1 and gRNA3, which target the TAL20 EBE site and the *MeSWEET10a* 5′UTR upstream of the start codon. Construct 250 contains gRNA4 that targets upstream of the TATA box and TAL20 EBE and gRNA5 that targets the TAL20 EBE downstream of the TATA box. Four rounds of cassava transformation with all 3 constructs were performed. In total, 24 transgenic lines were recovered with 7 mature lines generated from construct 108, 8 from construct 249, and 9 from construct 250. Leaf tissue was sampled from each line at the plantlet stage in tissue culture, and plants were genotyped by Sanger sequencing. Twenty-three out of 24 lines had edits within the *MeSWEET10a* promoter and/or coding sequence (Table 1, Supplementary Fig. S4A). One line was recovered containing only edits within the TAL20 binding site while maintaining an intact TATA box; however, this line died during the tissue culture process.

**Table 1.** Overview of *MeSWEET10a* mutant line genotypes

Mutant type	General description	Number of lines
WT-like transgenic	Transgenic lines with no edits at gRNA target sites	1
Silent mutant	Edits that do not impact coding sequence	13
TAL20 EBE promoter INDEL	Edits within the TAL20 EBE with intact TATA box	1^a^
*MeSWEET10a* promoter INDEL	Edits in the TATA box predicted to impact expression	11
*MeSWEET10a* frameshift	Edits after the TSS expected to impact coding sequence	3

Mutation type summary of the 29 transgenic lines recovered from all rounds of transformation based on Sanger sequencing results.

^a^A line that died during the tissue culture process.

The genotypes of 5 of these additional lines (Fig. 2, B and C) were confirmed using gDNA clone sequencing and/or whole genome resequencing (Supplementary Figs. S3, C and D, and S4B). From clone sequencing, line #27 has a 185 bp deletion spanning the TATA box, TAL20 binding site, and 5′UTR and a 1 bp frameshift insertion after the start codon. Line #30 has a 1 bp deletion upstream of the TATA box and a 2 bp frameshift deletion downstream of the start codon. Similar to line #269, it is not clear whether these lines are truly homozygous mutants, or if a second mutant allele exists but was not revealed through these analysis methods. Line #41 is a biallelic mutant with 1 allele that has an 11 bp deletion at the TATA box/TAL20 EBE site and a 1 bp insertion in the 5′UTR. The second allele has a 5 bp deletion at the TATA box/TAL20 EBE site and a 1 bp insertion in the 5′UTR. Line #54 is biallelic with 1 allele containing a 5 bp deletion at the TATA box/TAL20 EBE site and a 1 bp insertion in the 5′UTR. The second allele contains 76 bp deletion spanning the TATA box/TAL20 EBE site. Line #69A is a biallelic silent mutant with 1 allele that has a 1 bp deletion upstream of the TATA box and 1 insertion in the 5′UTR and another allele that has a 1 bp deletion upstream of the TATA box. gDNA from lines #27, #30, #41, #54, and #69A all produced a PCR product near 2.1 kb corresponding with their insertion/deletion types (Fig. 2D). An overview of the mutant types generated from all transformations is provided in Table 1. Results from select stages of the transformation pipeline are reported in Supplementary Table S1. Whole genome sequencing for lines #269, #338, #27, #30, #41, and #54 revealed the transgene insertion number and location for each line (Supplementary Fig. S5). Mutant plants were moved from tissue culture to soil and phenotypically characterized for height, node number, internode length, petiole length, central lobe length, central lobe width, and whole lobe length (Fig. 2E, Supplementary Table S2). Based on these characteristics, mutant plant traits were physiologically similar to wild-type cassava.

**Figure 3.**
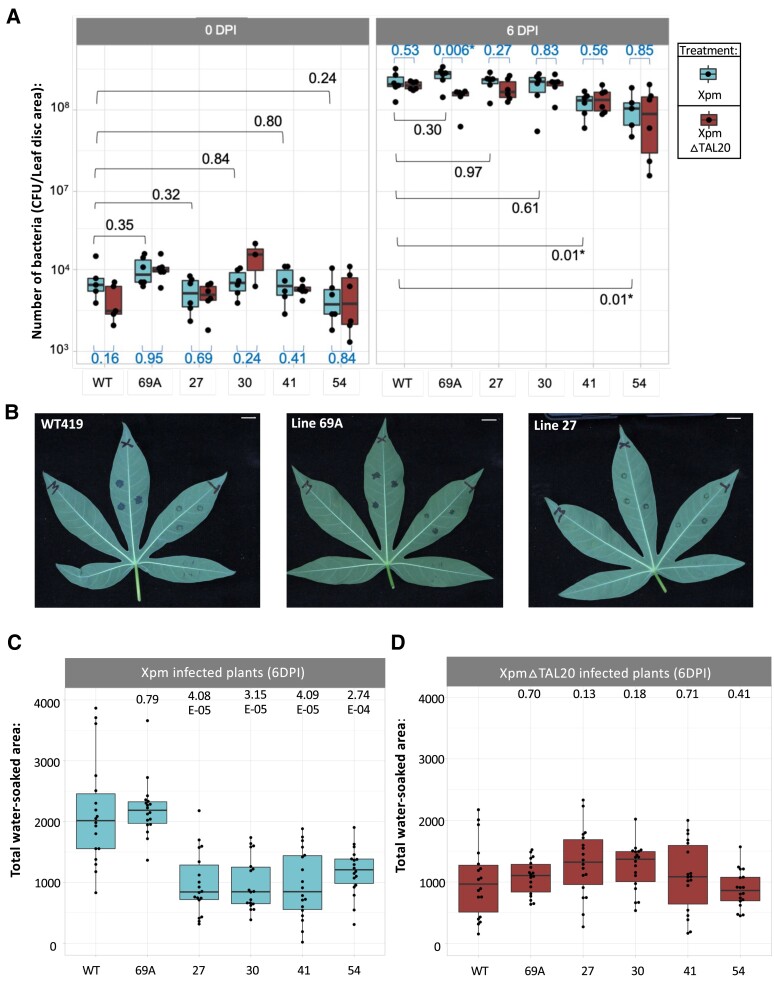
*MeSWEET10a* mutant line CBB disease symptoms post *Xpm* infection. **A)** Number of bacteria in cassava leaves measured at 0 DPI (left) and 6 DPI (right) with *Xpm* and Xpm△TAL20 treatments. CFUs (CFU/leaf disk area, *y* axis) are plotted by plant genotype (*x* axis) tested (wild type or mutant). Dots represent sample replicates from an independent bacterial growth experiment. **B)** Representative images of infected wild-type (left), silent mutant line 69A (middle), and mutant line 27 (right) cassava leaves detached from the plant and imaged at 4 DPI. X, *Xpm*; T, Xpm△TAL20; M, mock. Scale bar = 1 cm. **C)** Total water-soaked area (pixels, *y* axis) of *Xpm*-infected plants (genotypes, *x* axis) at 6DPI. **D)** Total water-soaked area (pixels, *y* axis) of Xpm△TAL20-infected plants (genotypes, *x* axis) at 6 DPI. Dots represent individual water-soaked lesions from 3 independent water-soaking assay experiments combined. In all boxplots, the calculated *P*-values (unpaired Student’s *t* test with unequal variance) are shown above or below each box plot. Dots outside whiskers represent outliers based on default settings of the R package ggplot2. The horizontal line within the box represents the median sample value. The ends of the boxes represent the 3rd (Q3) and 1st (Q1) quartiles. The whiskers show values that are 1.5 times interquartile range (1.5 × IQR) above and below Q1 and Q3.

As a rapid screen to identify mutants that avoid TAL20-mediated induction of *MeSWEET10a*, leaves were detached from plantlets in tissue culture and syringe infiltrated with wild-type *Xpm*, Xpm△TAL20, or mock treatments. Samples were collected at 48 HPI for RNA extraction and RT-PCR analysis (Supplementary Fig. S6). When infected with *Xpm*, but not Xpm△TAL20 or mock treatments, the expected 123 bp band corresponding to induction of *MeSWEET10a* was observed in wild-type plants and the silent mutant, line #69A. In contrast, no band was observed for samples from lines #27, #30, #41, and #54 (Fig. 2F). These results support the hypothesis that edits in these lines prevent induction of *MeSWEET10a* by TAL20. *Xpm* and Xpm△TAL20 were infiltrated into cassava leaves, and bacterial growth of both strains was similar in infected wild-type and *MeSWEET10a* mutant plants, consistent with our previous research (Veley et al. 2023; Fig. 3A, Supplementary Fig. S7). However, a visible difference in water-soaking lesions was observed (Fig. 3B, Supplementary Fig. S8), and these lesions were quantified using a machine learning image analysis method (Elliott et al. 2022). Lines #27, #30, #41, and #54 all exhibited reduced water-soaked lesion area compared to wild-type cassava and line #69A, after challenge with *Xpm* (Fig. 3C). There was no significant difference in Xpm△TAL20 lesion area between any of the mutant lines compared to wild-type plants (Fig. 3D). Similar results were obtained from line #269 and #338 mutant plants infected with *Xpm* and Xpm△TAL20 (Supplementary Fig. S8). Therefore, we conclude that *MeSWEET10a* mutant plants have decreased susceptibility to CBB, as measured by disease lesion severity.

**Figure 4.**
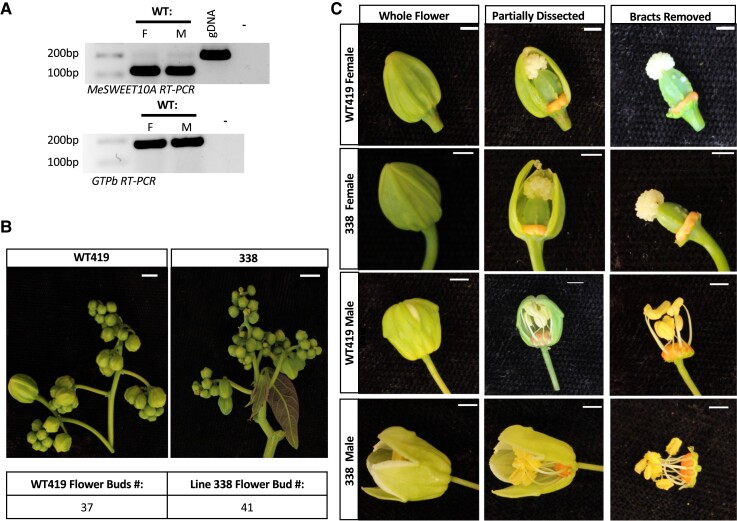
Characterization of *MeSWEET10a* mutant flower morphology and reproductive viability. **A)** RT-PCR of WT419 female (F) and male flowers (M) collected from field-grown plants. Top gel shows results of RT-PCR with primers amplifying *MeSWEET10a* with an expected product size of 123 bp. The bottom gel shows results of RT-PCR with primers amplifying the housekeeping gene GTPB as a control for sample loading with an expected product size of 184 bp. gDNA is from WT419 leaf tissue included as a positive control and “-” denotes a negative water control. **B)** Representative images of WT419 (left) and line 338 (right) inflorescence structures detached from individual field-grown plants for imaging (top). The number of flower buds present on each inflorescence is presented in table format (bottom). Scale bar = 5 cm. **C)** Representative female and male flowers collected from WT419 and line 338 flowering field-grown plants. Images of the same flower were taken as whole flowers (left) partially dissected with 1 or 2 petal-like bracts removed (middle) and dissected with all petal-like bracts removed (right). Scale bar = 0.25 cm.

Unlike leaves, cassava flowers have endogenous expression of *MeSWEET10a* (Fig. 4A; Veley et al. 2021). Thus, we wanted to determine if mutating *MeSWEET10a* would impact flower development or reproductive capability. Cassava plants do not readily flower and set seed in greenhouse or growth chamber conditions. However, *MeSWEET10a* mutant lines #338 and #54 had been established at a field site in Hilo, Hawaii, along with wild-type cassava plants (WT419, Nase3, TME7, and 60444). Line #338 plants were the first MeSWEET10a mutants to flower in the field. Ten months after transplanting, line #338 formed the first inflorescences of male and female flower buds (Fig. 4B). WT419 and line #338 female and male flowers were collected, and the petal-like bracts were dissected (Perera et al. 2012). No obvious visible differences were observed between line #338 and WT419 flower structures (Fig. 4C). Line #338 female and male flower buds were collected for RNA extraction and RT-PCR. The *MeSWEET10a* RT-PCR product was detected in both WT419 and line #338, demonstrating that the mutations in line #338 do not block transcription although translation of these RNA molecules would likely not produce a functional protein (Supplementary Fig. S9).

**Figure 5.**
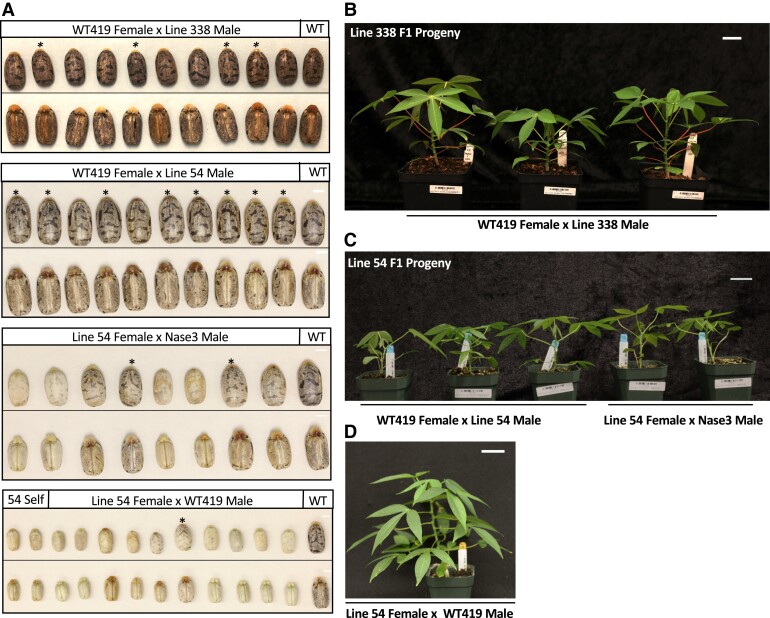
Characterization of MeSWEET10a mutant reproductive viability. **A)** Topside and underside images of 10 seeds from WT419 female × line 338 male crosses along with a WT419 seed (first image set), 10 seeds from WT419 female × line 54 male crosses along with a WT419 seed (second image set), 8 seeds from line 54 male × Nase3 male crosses along with a WT419 seed (third image set), 2 seeds from line 54 self-crosses, and 10 seeds from line 54 female × WT419 male crosses along with a WT419 seed (fourth). Scale bar = 0.25 cm. The same WT419 seed was used in the second and third image sets. Asterisks (*) denote F1 seeds that germinated. **B)** Image of 3 WT419 female × line 338 male F1 individuals that germinated postplanting in soil. Scale bar = 3.0 cm. **C)** Image of 3 WT419 female × line 54 male F1 individuals (left) and 2-line 54 female × Nase3 male F1 individuals that germinated postplanting in soil (bottom). Scale bar = 3.0 cm. **D)** Image of a line 54 female × WT419 F1 individuals that germinated postplanting in soil. Scale = 3.0 cm.

Line #54 plants were introduced to the field later than line #338 and began producing flowers 9 mo after transplanting. Both lines #338 (promoter and coding sequence mutant) and #54 (promoter mutant) were used for crosses to examine flower reproductive viability and compared to WT419 self-crosses. From 97 WT419 self-crosses, a total of 152 F1 seeds were recovered (Supplementary Fig. S10A). All 110 crossing attempts using line #338 female flowers failed to produce viable seed. However, 171 crossing attempts using line #338 male flowers successfully resulted in 97 F1 seed. One hundred and twenty-nine crossing attempts with line #54 female flowers resulted in 24 seed, 2 of which came from self-crosses. Sixty-eight crosses between WT female and line #54 male flowers resulted in 37 seed. F1 seed length and weight were measured (Supplementary Fig. S10, B and C, and Table S3). Seed derived from crosses with a WT419 female and line #338 or line #54 male all appeared similar (Fig. 5A). However, seeds from crosses with a line #54 female were a lighter color and had a lower average length and weight compared to WT419 self- or open-pollinated seed. One common way of measuring seed viability is through float tests; seeds that sink are expected to germinate while seeds that float commonly do not (Pegman et al. 2017). Float tests were performed for all F1 seeds (Supplementary Table S3). Additionally, germination tests were performed on a select number of seeds. PCR and Sanger sequencing–based genotyping was completed on a batch of germinated F1 to confirm mutant parent type (Supplementary Fig. S11). The germination rate for WT419 open-pollinated and selfed seed was 87% (Supplementary Table S3). The germination rate for line #338 F1 seed was 47%. The overall germination rate for line #54 F1 seed was 47.8%. Due to the observed differences in line #54 female- and male-derived seeds, the germination rate for each seed type was calculated, as well. Line #54 female-derived seed germination was 14% whereas male-derived seed had a germination rate of 86%. Neither of the 2 line #54 self-crossed seeds germinated. However, 2 F1 seeds from line #54 × Nase3 and 1 F1 seed from line #54 × TME419 germinated to produce healthy looking plants (Fig. 5, B to D).

## Discussion

Previous work in other systems has demonstrated that gene editing can be used to block pathogen access to host susceptibility genes. In this study, we sought to use gene editing to create cassava plants with reduced susceptibility to *Xpm*-induced CBB. Specifically, we targeted the *MeSWEET10a* susceptibility gene that is ectopically induced by the *Xpm* effector protein, TAL20, during infection. Based on previous work in cassava and rice, we speculated that native function of *MeSWEET10a* may prove essential to flower development. Therefore, we designed a dual gRNA CRISPR/Cas9 strategy to generate diverse *MeSWEET10a* mutant lines with edits at the TAL20 EBE binding site and/or the *MeSWEET10a* coding sequence. Mutants that lacked TAL20-mediated induction of *MeSWEET10a* had significantly reduced disease symptoms. Further characterization through crossing experiments indicate that *MeSWEET10a* expression may be essential in female, but not male, flowers and that specific edits within the promoter sequence may be able to block TAL20 induction while still producing viable F1 seed.

In this paper, mutant and wild-type plants were phenotyped for CBB disease severity through bacterial growth assays and water-soaked lesion analysis. While a consistent difference in bacterial titer was not observed, we found that *MeSWEET10a* mutant lines had significantly reduced water-soaked lesions post *Xpm* infection compared to infected wild-type cassava. The exact mechanics of how *MeSWEET10*a is used by *Xpm* to promote CBB and pathogen virulence remains unknown. *MeSWEET10*a is a clade III SWEET gene that is presumed to export sucrose and glucose from the plant cell into the apoplast where *Xpm* proliferates. One hypothesis is that *Xpm* uses these sugars as a carbon source. However, if the *MeSWEET10a* exported sugars were a direct carbon source for *Xpm*, we would expect that loss of TAL20 would significantly impact bacterial growth. In a previous study, a decrease in bacterial growth was observed for Xpm△TAL20 compared to wild-type *Xpm*. This previous study used a different genotype of cassava and a modified bacterial growth assay (Cohn et al. 2014). In the current work, we did not observe a consistently significant difference between *Xpm* and Xpm△TAL20 colony-forming units (CFUs). Yet, whenever a bacterial growth difference was observed, Xpm△TAL20 titer trended downward compared to *Xpm*. This may indicate that *MeSWEET10a* has a minor effect on bacterial growth but that the effect is below the limit of sensitivity of the bacterial growth assays and conditions used in our study.

An additional hypothesis is that *MeSWEET10a* exported sugars may serve as an osmolyte for *Xpm*. As sucrose and glucose are exported out of the plant cell, there is also an osmotic movement of water. *MeSWEET10*a mutants have consistently reduced water-soaked lesions after infection with *Xpm* compared to infected wild-type cassava. Water-soaked lesions are dark angular spots that occur during pathogenesis as water is moved from the plant cell into the apoplast (Schwartz et al. 2017). Many plant pathogens induce water-soaked leaf lesions during early stages of plant infection (Aung et al. 2018). Other studies have suggested that the role of water soaking is to create an aqueous environment to aid in bacterial colonization from the plant surface into the apoplast or to help with bacterial spread once in planta (Xin et al. 2016). Perhaps the efflux of sugar and water into the apoplast increases bacterial entry into the plant which would not be captured through syringe infiltration–based infection assays. Additionally, *Xpm* eventually spreads throughout the plant vasculature after initial colonization at the surface of mesophyll cells. It is possible that induction of *MeSWEET10a* by TAL20 may play a role in bacterial spread. Another study in the *Xanthomonas gardneri–*pepper pathosystem reported that reduced water-soaked lesion symptoms did not correspond to a decrease in bacterial growth (Schornack et al. 2008). In the future, additional work is required to tease apart the role of *MeSWEET10a* exported sugars and water soaking in *Xpm* pathogenesis, and most importantly, how these disease phenotypes translate to observed disease under field conditions.

Through this work, several features of gene editing in a crop like cassava were highlighted. For example, we note that while the disease phenotypes among the mutant lines that avoided *MeSWEET10a* induction by TAL20 were consistently less severe than wild-type plants, these phenotypes were somewhat variable. This may reflect somaclonal variation from the cassava tissue culture process. Given how heterozygous cassava is, we expect to see similar variation in F1 progeny, as these lines will segregate many polymorphic loci. In F1 progeny in which a wild-type allele has been crossed in, we may also expect to see new alleles arise, if the Cas9 machinery continues to express and enable Cas9-mediated target cleavage. While most mutant lines showed biallelic mutations, in a few cases, a second mutant allele was not observed. Given that the 2 alleles of *MeSWEET10a* would be cut and repaired independently, it is highly unlikely that homozygous mutations would be recovered. Thus, the failure to observe a second allele in these plants more likely indicates the limitations of our genotyping strategies.

SWEET genes have been implicated in various roles in plants such as nectar secretion, pollen development, seed filling, and phloem loading (Feng and Frommer 2015). In cassava, the native function of *MeSWEET10a* remains unknown. We inspected the impact of editing *MeSWEET10a* on cassava flowers as the flowers have endogenous expression of the gene (Veley et al. 2021). No obvious visible morphological defects were observed in *MeSWEET10a* mutants compared to wild-type flowers. However, crosses conducted with *MeSWEET10a* mutant lines and wild-type plants revealed differences in female and male flower reproductive capability. In line #338, an *MeSWEET10a* mutant with promoter and coding sequence edits, only crosses with male flowers produced viable F1 seeds. Crosses with line #338 female flowers failed to produce F1 seed. In line #54, an *MeSWEET10a* mutant with promoter edits at the TATA box and TAL20 EBE, crosses with both male and female flowers resulted in F1 seed. However, line #54 female-derived seeds were generally smaller and had reduced germination compared to male-derived seeds. We note that the 3 seeds derived from line #54 female flowers that did germinate were the seeds that were phenotypically most similar to wild-type–derived seeds. Regardless, it is not yet clear whether the mutations in line #54, that alter the TATA box, have a negative impact on flower development. Examples exist wherein the TATA box was shown to be dispensable for gene expression. In the case of *MeSWEET10a*, this will need to be further experimentally investigated. Cassava is a highly heterozygous plant with a high genetic load (Mansfeld et al. 2021; Ramu et al. 2017). As such, self-crosses and further inbreeding often yield phenotypically weak plants. Our results suggest that MeSWEET10a may play a role in female but not in male flower reproductive viability. However, these data may also reflect some endogenous trait segregation. Future investigation is needed to determine the full function of MeSWEET10a in flowers. For example, microscopy comparing the structure of *MeSWEET10a* mutant and wild-type cassava flowers could determine if there are differences in flower development not visible to the naked eye. Microscopy could also be used to compare *MeSWEET10a* mutant and wild-type fruit and F1 seed. In cassava, female flowers form round fruit a week after successful pollination and each fruit can contain between 1 and 3 seeds. The fruit fully develops about 3 mo post pollination, and seeds can then be harvested (Kawano 1980). Unsuccessful crosses can fail to develop fruit, or the fruit can abort sometime after initial formation. In line #338 female flowers, fruit was able to form post pollination, but they eventually aborted. Understanding when line #338 fruit/seed development fails may help to reveal the role MeSWEET10a plays in female flowers.

The ideal *MeSWEET10a* mutant line, for CBB resistance, would contain edits at the TAL20 EBE site while maintaining an intact TATA box and gene coding sequence for native plant function. In this study, 1 line with EBE only edits and an intact TATA box was recovered, and this line died during the tissue culture process. It is possible that this outcome is selected against for an unknown reason. More likely, if many additional lines were recovered, we would eventually achieve this outcome. We note that the 14 *MeSWEET10a* mutants (3 knockouts and 11 with promoter mutations) that we recovered represent several years of work and many independent transformations. While resources for research on cassava have increased dramatically over the last decade, recovery of transgenic lines remains a bottleneck. This contrasts with some other systems where hundreds or even thousands of independent transgenic lines can be recovered in as little as half a year. The cassava community is one of the many communities eagerly awaiting breakthrough technologies that improve transformation efficiency and/or shorten the associated timelines. One factor complicating the ability to generate EBE-specific edits to the *MeSWEET10a* TAL20 binding site is overlap between the TATA box and EBE. In cassava, other TAL effectors localize to EBE sites that include TATA box motifs (Cohn et al. 2016). Other work shows that EBE overlap within or localization near the host TATA box is common in TAL effector S gene target sites (Grau et al. 2013; Pereira et al. 2014; Pérez-Quintero et al. 2015). Alternative gene editing strategies including base editing, use of different endonucleases such as Cas12a, or the use of a single CRISPR/Cas9 gRNA at the TAL20 EBE downstream of the TATA box may increase the chances of recovering *MeSWEET10a* mutants with EBE only edits (Paul and Montoya 2020; Azameti and Dauda 2021).

Editing 1 S gene alone may not be sufficient to significantly reduce cassava susceptibility to CBB in a field setting. Additionally, other S genes may play a more direct role in *Xpm* growth in cassava. Thus, investigating the impact of additional S genes on *Xpm* virulence and stacking edits at different S gene targets may be required to develop cassava plants with sustained resistance to CBB. The findings in this study suggest that blocking *MeSWEET10a* induction decreases cassava susceptibility to CBB. Furthermore, we found that promotor and coding sequence edits in *MeSWEET10a* differentially impact female flower reproductive viability. We are optimistic that an ideal promoter mutation would leave endogenous expression of *MeSWEET10a* intact while blocking TAL effector binding. Overall, this study serves as a good foundation and road map for the development of cassava with improved CBB tolerance.

## Materials and methods

### Construct design and cloning

The *MeSWEET10a* (Manes.06G123400) FASTA sequence file was downloaded from Phytozome (cassava [*M. esculenta*] genome v6.1) and uploaded to the software Geneious. Notable promoter regulatory elements were annotated as previously reported by Cohn et al. (2014) including the EBE site where TAL20 binds. The reported EBE sequence was confirmed using the TALEnt target finder tool. The Geneious “find CRISPR sites” function was used to find all potential targets and Cas9 (*Streptococcus pyogenes*)-specific PAM sites (sequence: 5′-NGG-3′). Candidate gRNA target sequences were selected based on those whose targets were near the TAL20 EBE and the translational start site of *MeSWEET10a* or within the 5′UTR. Candidate gRNAs were further analyzed by comparing the gRNAs against the cassava genome to identify potential off-targets using NCBI-BLAST. All constructs were assembled using a multiple gRNA spacer Csy4 array as previously described, and Cas9 was expressed by a 35 s promoter (Čermák et al. 2017). Three constructs were used for this study. All constructs were designed to carry 2 gRNAs, were cloned in the pTRANS_220D backbone, and have a kanamycin resistance cassette. Construct 108 carries gRNA1 (GAGAAGCGTTTATATAGGGG) that targets the TAL20 EBE site and gRNA2 (GAAGTCCAATGACAAGTGCA) targeting the *MeSWEET10a* translation start site. Construct 108 also carries an intended EBE repair template (as an attempt to replace the sequence) containing homology arms that flank the EBE (1,079 bp 5′ homology arm and 727 bp 3′ homology arm). Construct 249 was designed to carry the TAL20 EBE site target gRNA1 (GAGAAGCGTTTATATAGGGG) and gRNA3 (ACTCTCTTTCCCTTGTGCAG), which target the 5′UTR with no repair template. Construct 250 was designed to carry gRNA4 (AAAATATGTCAATGTAACAG) and gRNA5 (TATGTTGTGCAATGATGGAT), which target the 5′UTR and EBE with no repair template. Construct assembly was confirmed through colony PCR, Sanger sequencing, and by Illumina sequencing. Constructs were transformed into LBA4404 *Agrobacterium* cells for cassava transformations. All construct sequences, maps, and Illumina reads are available in the Supplementary data.

### Plant materials and growing conditions

Transgenic cassava lines expressing the CRISPR/Cas9 machinery and gRNAs were generated in the cassava cultivar TME419 (WT419) through *Agrobacterium*-mediated transformation as described (Chauhan et al. 2015). Transgenic FEC cells were selected for resistance using 100 mM paromomycin (275 *μ*L/L) on spread plates. A total of 100 mM paromomycin (450 *μ*L/L) was used to further select for resistant transgenic cells on stage 1, 2, and 3 plates. Transgenic FEC cells and eventual transgenic plantlets were maintained in tissue culture in conditions set to 28°C ± 1°C, 75 *μ*mol m^−2^ s^−1^ light, and 16-h light/8-h dark. Plantlets were transferred to soil on a misting bench and covered with domes to maintain high humidity. After establishment in soil, plants were moved from the misting bench and acclimated to greenhouse conditions set to 28°C, 50% humidity, 16-h light/8-h dark, and 1,000-W light fixtures that supplemented natural light levels below 400 W m^−2^. Following bacterial infection assays, plants were kept in a posttreatment growth chamber with conditions set to 27°C, 50% humidity, and 12-h light/12-h dark. F1 seeds generated from crosses were planted in soil and kept in a plant growth chamber set to 37°C, 60% humidity, and 12-h light/12-h dark at 400 *μ*mol m^−2^ s^−1^. Once seedlings germinated, they were transferred to larger pots and moved to greenhouse conditions listed above.

### DNA extraction and transgenic line genotyping

As an initial pass, transgenic lines recovered from the first transformation with construct 108, a PCR followed by restriction digest and gel electrophoresis strategy was used. Mutants with varying HaeIII digest patterns were suspected to have deletions and were moved forward for Sanger sequencing with full-length PCR product. In subsequent rounds of transformation, mutants with both point mutations and insertions/deletions were further characterized. For later transformations, leaf lobe samples from transgenic lines were collected from 2 to 3 individual plantlets and pooled into 2-mL Eppendorf Safe-Lock tubes with 3 disposable 3-mm Propper solid glass beads. The sample tubes were flash frozen in liquid nitrogen and ground to a fine powder using a QIAGEN TissueLyser II machine at 30 Hz for 3 min until the sample was fully homogenized. gDNA was extracted using the Sigma GenElute Plant Genomic DNA Miniprep Kit. The *MeSWEET10a* region of interest was amplified using “outer” primers designed to avoid amplification of the EBE repair template present in the construct 108 transgene, and a 2.1 kb product was generated for each line. All primers used in this study are provided in Supplementary Table S4. The PCR product was purified using the Qiagen QIAquick PCR Purification kit. The samples were sent for Sanger sequencing using secondary “inner” primers designed to start amplification closer to the gRNA target sites. Transgenic line trace files were compared to wild-type TME419 trace files, and edits within and across each gRNA were identified using the Geneious bioinformatics tool. To determine if edits were homozygous for each allele, clone sequencing was performed on select lines. For each line, gDNA was extracted, the *MeSWEET10a* region was amplified by PCR, and the PCR products were cloned into the PCR4-TOPO TA vector using the Thermo Fisher TOPO TA Cloning Kit. Colonies were selected and confirmed by restriction digest. Plasmid DNAs from colonies positive for the *MeSWEET10a* amplicon were extracted, and multiple colonies per line were sent for either Sanger sequencing (lines #269 and 338) or Oxford Nanopore sequencing (lines #27, 30, 41, 54, and 69A). In cases where additional sequencing was needed to confirm mutant edits, whole genome sequencing was used as described below.

### Whole genome sequencing for identification of transgene location(s) and confirming select *MeSWEET10a* mutant line edits

For each construct, a custom reference genome was created, which contained the haplotype-resolved genome assembly for cassava variety TME204 (Qi et al. 2022) along with the vector sequence from the T-DNA left border through the T-DNA right border of the appropriate construct. The program bwa mem (version 0.7.12-r1039) was used to align the whole genome sequencing data to the custom reference genome (Li 2013). Illumina reads where 1 pair aligned to the T-DNA insertion sequence and the other aligned to the cassava genome were isolated using samtools (version 1.11; Danecek et al. 2021) and used as input for de novo assembly by Trinity (version v2.1.1; Grabherr et al. 2011). Resultant contigs were then used in a blastn (version 2.12.0+) query against a BLAST database of the custom reference genome initially used for bwa alignments (Sayers et al. 2022). Contigs where a portion matched the cassava genome and another portion matched the T-DNA insertion sequence identified the coordinates of the 5′ and 3′ ends of an insertion point within the genome. Integrative Genomics Viewer (IGV; version 2.12.3) was used for manual inspection and visualization of the aligned WGS data to the custom T-DNA insertion plus genome (Thorvaldsdóttir et al. 2013). To confirm the genotype of select *MeSWEET10a* mutant lines, Illumina, paired end, and whole genome sequencing reads from each haplotype of *MeSWEET10a* (Manes.06G123400) were isolated and used for de novo assembly to identify the edits generated.

### Bacterial inoculations

*Xanthomonas* strains were struck from glycerol stocks onto NYG agar plates containing appropriate antibiotics. The strains used were Xpm668 (rifampicin 50 *µ*g/mL), Xpm668ΔTAL20 (suicide vector knockout, tetracycline 5 *µ*g/mL, rifampicin 50 *µ*g/mL), Xe85-10 (rifampicin 50 *µ*g/mL), and Xe85-10+TAL20_Xpm668_ (rifampicin 50 *µ*g/mL, kanamycin 50 *µ*g/mL; Cohn et al. 2014). *Xanthomonas* strains were grown in a 30°C incubator for 2 to 3 d. Inoculum for each strain was made by transferring bacteria from plates into 10 mM MgCl_2_ using inoculation loops and brought up to a concentration of OD_600_ = 0.01 for bacterial growth and water-soaked lesion assays and OD_600_ = 1 for RT-PCR. Leaves on cassava plants were inoculated using a 1.0-mL needleless syringe. For each replicate assay, 2 cassava plants per background (WT or transgenic) were used for inoculations, and 4 leaves were inoculated on each plant. One bacterial strain suspended in 10 mM MgCl_2_ was inoculated per leaf lobe with 3 injection sites, and mock inoculations of 10 mM MgCl_2_ alone were included. In total, there were 9 infiltrated sites per leaf.

### RNA extraction and RT-PCR analysis

For lines with edits of interest, RNA extraction and RT-PCR were performed at the plantlet stage in tissue culture and on soil established plants in the greenhouse. At the plantlet stage, 9 leaves were detached from every transgenic line and 3 leaves each were syringe infiltrated with either mock (10 mM MgCl_2_ alone) or *Xanthomonas* (Xpm668) on sterile petri dishes. For each line, a set of 3 infiltrated leaves per treatment were kept on MS2 plates in a posttreatment room light shelf. At 48 HPI, samples were collected, and 3 infiltrated leaves per treatment were pooled into Eppendorf Safe-Lock tubes with 3-mm glass beads. For greenhouse plants, 1 leaf was selected (3 biological replicates per plant background) and syringe infected with 3 infiltrated sites per treatment (mock [10 mM MgCl_2_ alone] or *Xanthomonas* [*Xpm668*] +/− TAL20 strains, or *Xe* [Xe85-10] +/− TAL20 strains) on separate leaf lobes. At 48 HPI, leaf punches from the infiltrated sites were collected using a size 7-mm core borer and technical reps were pooled into Eppendorf Safe-Lock tubes with 3-mm glass beads. Samples were flash frozen in liquid nitrogen and ground using TissueLyser settings described above. Total RNA was extracted from each sample using the Sigma Spectrum Plant Total RNA Kit. One microgram of RNA was DNase treated using Promega RQ1 DNase enzyme and reverse transcribed into cDNA using Thermo Fisher Scientific SuperScript III Reverse Transcriptase. RT-PCR was performed on each sample using primers specific to *MeSWEET10a* and to cassava *GTPb* (Manes.09G086600) as a constitutively expressed control. All primers used in this study are provided in Supplementary Table S4. RT-PCR results were analyzed to identify transgenic lines in which ectopic expression of *MeSWEET10a* was not induced by *Xanthomonas* (+TAL20) infection as is normally seen in wild-type cassava infected with *Xanthomonas* (+TAL20).

### Bacterial growth assay

Cassava leaves were infiltrated with either 10 mM MgCl_2_ (mock control) or *Xanthomonas* (*Xpm668* strains +/− TAL20) suspended in 10 mM MgCl_2_ as described above. Leaf punch samples were taken at the site of infiltration using a 7-mm core borer (size 4) at 0, 2, 4, and 6 d post inoculation (DPI). For day-0 samples, infiltrated spots were allowed to dry down prior to processing. Individual leaf punches were transferred to 2-mL Eppendorf Safe-Lock tubes with 200 *μ*L of 10 mM MgCl_2_ and 3 disposable 3-mm Propper solid glass beads. Samples were ground with a QIAGEN TissueLyser at 28 Hz for 3 min. Two hundred microliters of the ground sample was transferred to the first column of a labeled 96-well plate. Serial dilutions were performed by transferring 20 *μ*L of the nondilute sample (10^1^) to the next well containing 180 μL of 10 mM MgCl_2_. Samples were serially diluted to 10^4^ for day 0, 10^6^ for days 2 and 4, and for 10^8^ day 6. Ten microliters of each serial dilution was pipetted and spread onto labeled quadrants of an NYG plate with cycloheximide and the appropriate antibiotics for the infiltrated bacterial strain. Plates were incubated at 30°C for 2 to 3 d, and the number of colonies was counted. CFUs reported in this manuscript were transformed by sample area (CFU/leaf disk area where disk area = 0.38 cm).

### Water-soaked lesion imaging and quantification

Cassava leaves were detached and imaged at 0, 6, and 9 DPI. One leaf for every plant was collected for a total of 2 leaves per plant background at each time point. Line 338 and 269 leaves were imaged from above using a Raspberry Pi Sony IMX219 camera in an enclosed box with an overhead light. To increase image resolution, all subsequent infected plant leaves were imaged from above using a Canon EOS Rebel T5i camera with a 15- to 85-mm lens in an enclosed box with an overhead light. Images were processed and analyzed for water-soaked lesion area and grayscale color using a previously described custom machine learning image analysis tool developed for CBB disease quantification (Elliott et al. 2022).

### Flower inflorescences and flower bud imaging and dissection

Flower inflorescences and individual buds were detached from cassava plants (WT419 or line 338) growing in a field site (Hilo, Hawaii, USA). All flower inflorescences and individual buds were imaged in the field from above using a Canon EOS Rebel T5i camera with a 15- to 85-mm lens in a portable, partially enclosed pop-up light box with built-in LED lights controlled by a USB power pack. Images were postprocessed using Photoshop for color correction, and a scale bar was added using ImageJ version FIJI.

### F1 seed imaging

F1 seeds were imaged prior to planting using a Canon EOS 2DR with a 100-mm lens.

### Accession numbers

Sequence for *MeSWEET10a* is available at Phytozome (gene ID: Manes.06G123400).

## Supplementary Material

kiae243_Supplementary_Data

## Data Availability

The authors confirm that experimental data are available and accessible via the main text and/or the supplemental data. All raw data, R scripts, and water-soaked lesion images are available on figshare: 10.6084/m9.figshare.22718680.

## Supplementary data

The following materials are available in the online version of this article.

**Supplementary Figure S1.** Construct 108 transgenic lines’ restriction digest screen.

**Supplementary Figure S2.** Transgenic line sequencing from the first construct 108 transformation.

**Supplementary Figure S3.** Whole genome sequencing to confirm MeSWEET10a mutant line edits.

**Supplementary Figure S4.** Constructs 108, 249, and 250 Sanger sequencing data from 4 replicate transformations.

**Supplementary Figure S5.** Transgene insertion analysis.

**Supplementary Figure S6.** *Xpm* infected detached leaf RT-PCR.

**Supplementary Figure S7.** Additional replicates of bacterial growth assays.

**Supplementary Figure S8.** Lines 269 and 338 water-soaked lesion assay.

**Supplementary Figure S9.** RT-PCR of wild-type and line 338 flowers.

**Supplementary Figure S10.** *MeSWEET10a* mutant field cross results and F1 seed traits.

**Supplementary Figure S11.** Genotyping line #54 *MeSWEET10a* mutant F1 progeny.

**Supplementary Table S1.** Transformation results.

**Supplementary Table S2.** Plant morphology measurements.

**Supplementary Table S3.** Seed measurements and germination.

**Supplementary Table S4**. Primer list.
