# Supplementary material for: CRISPR/Cas9-generated mutations in a sugar transporter gene reduce cassava susceptibility to bacterial blight
Source: Plant Physiol. 2024 May 3;195(4):2566–78. doi: 10.1093/plphys/kiae243 (PMC11288762; doi:10.1093/plphys/kiae243)
Supplement: kiae243_Supplementary_Data [file kiae243_supplementary_data.zip › Supplementary Data.pdf]

A.

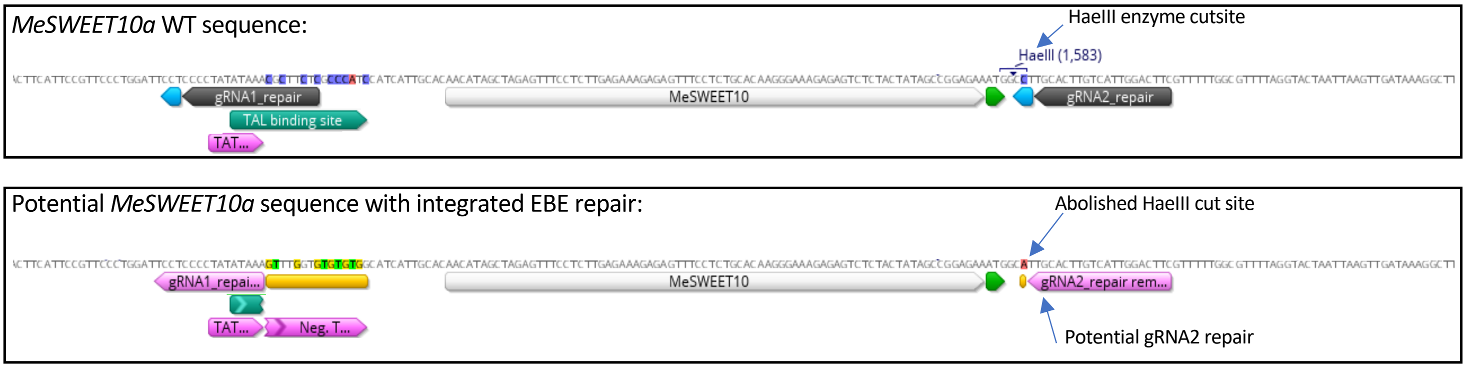

B.

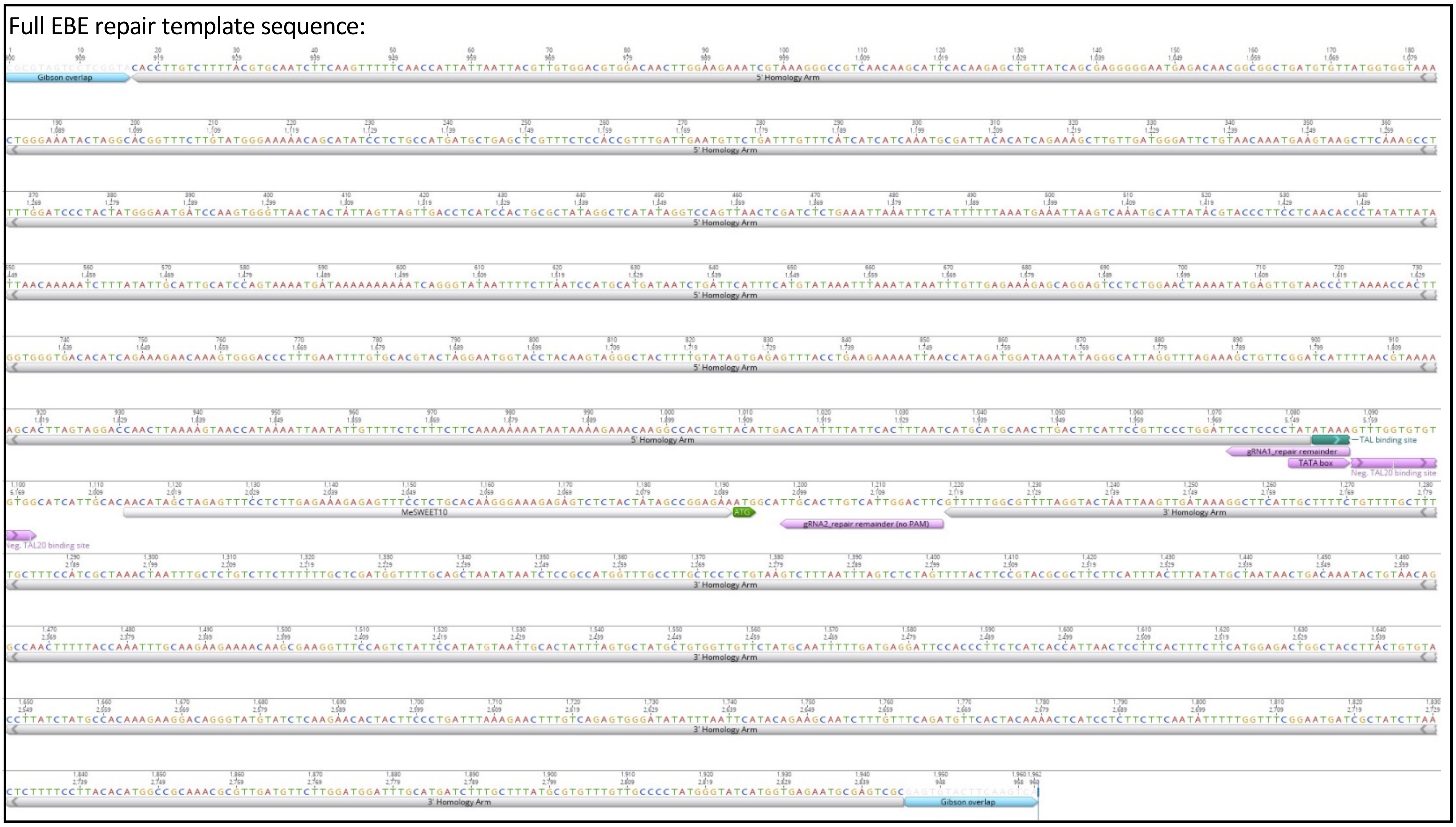

C.

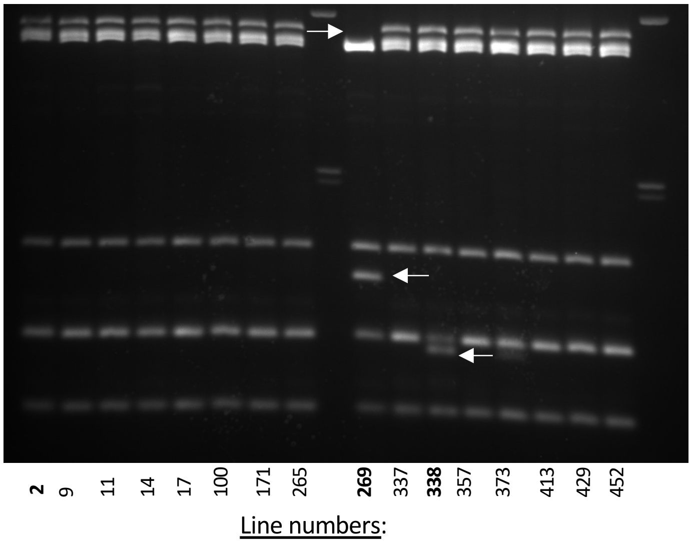

Supplementary Figure S1: Construct 108 transgenic lines restriction digest screen

A) Overview of HaeIII restriction digest strategy for *MeSWEET10a* region of interest in WT (top) and potential mutant with the EBE template repair (bottom). WT-like sequences were expected to remain uncut. However, mutants with integration of the repair template were expected to have an abolished HaeIII site and remain uncut. Blue arrows point to the HaeIII cut site and potential gRNA2 repair site from the template integration by homology-directed repair. **B)** Annotated Genious screenshots of the full EBE repair template sequence included in construct 108. 5' homology arm is ~1,079 base pairs and the 3' homology arm is ~727 base pairs. **C)** Example of an HaeIII digest performed on sixteen transgenic lines. White arrows point to digest patterns unlike the WT digest pattern. The expected digest pattern for WT is bands at 895, 384, 270, 271, 195, and 192 bp. Bolded line numbers (2, 269, and 338) were moved forward for Sanger sequencing.

A.

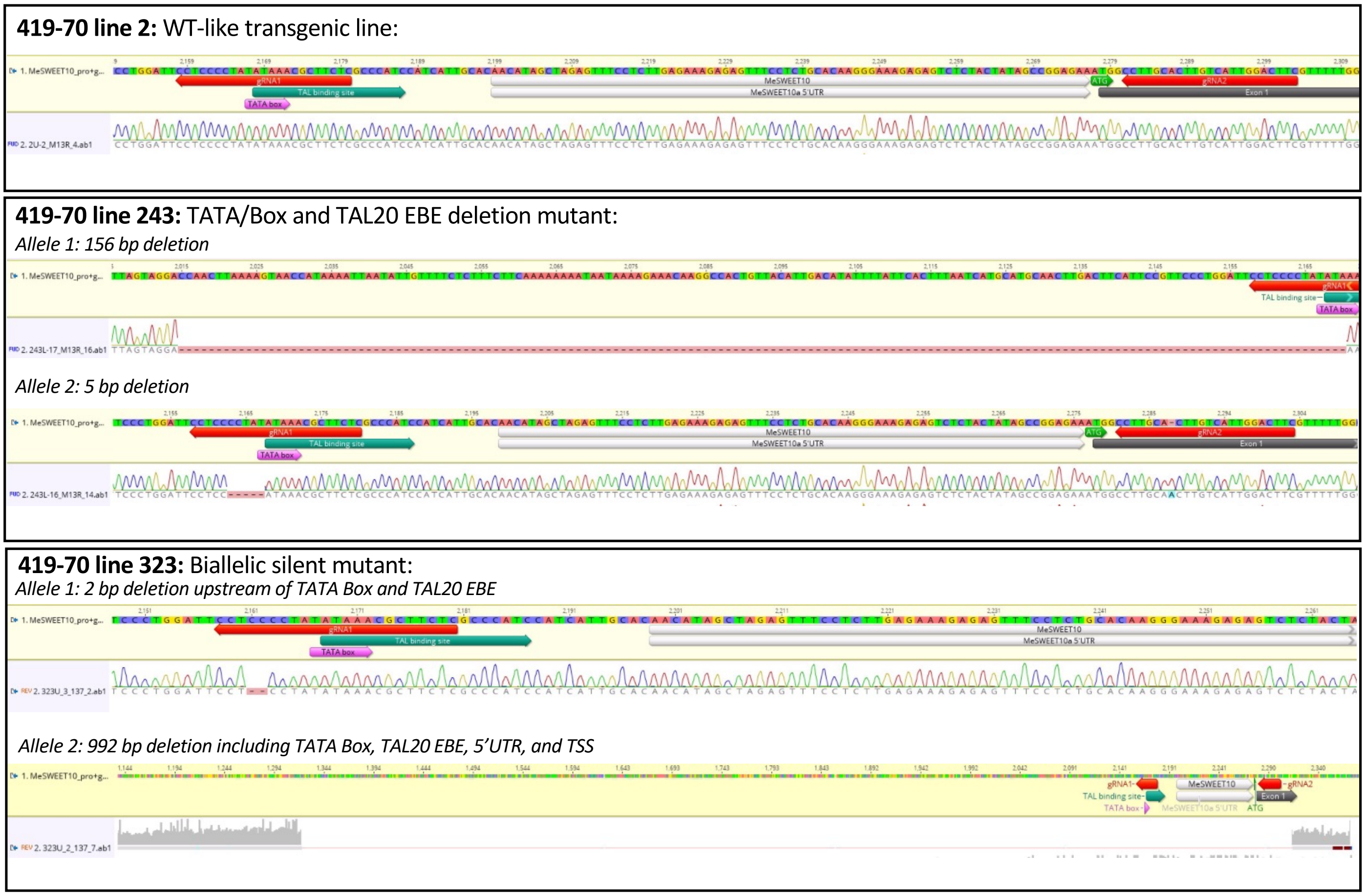

B.

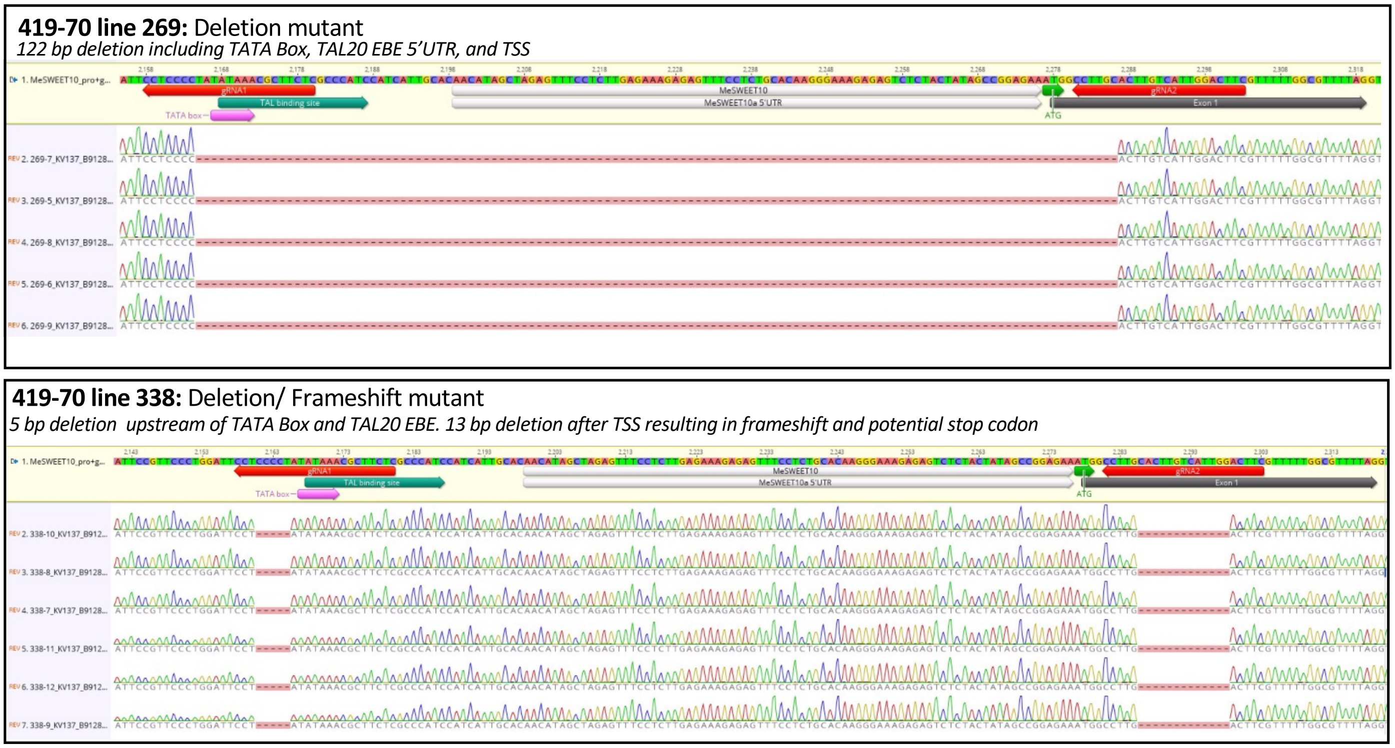

Supplementary Figure S2: Transgenic line sequencing from first Construct 108 transformation
A) Geneious screenshots showing Sanger-sequencing results for lines 2, 243, and 323. Mutation types and INDELs are described in text. **B)** Geneious screenshots of Sanger-sequencing results of *E. coli* clones containing *MeSWEET10a* gDNA from lines 269 and 338 plants.

A.

KE338 haplotype 1

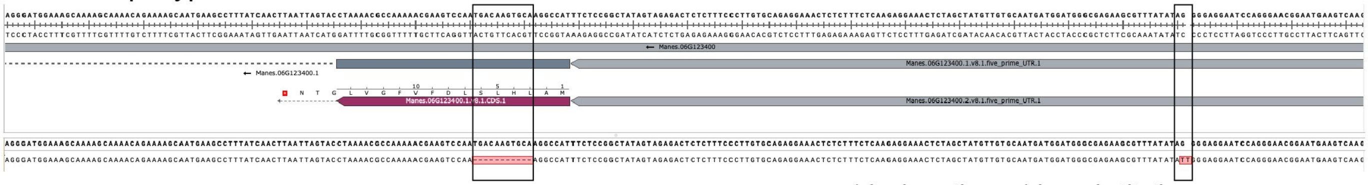

11 bp Deletion: TGACAAGTGCA

1 bp insertion + 1 bp substitution AG→ATT

KE338 haplotype 2

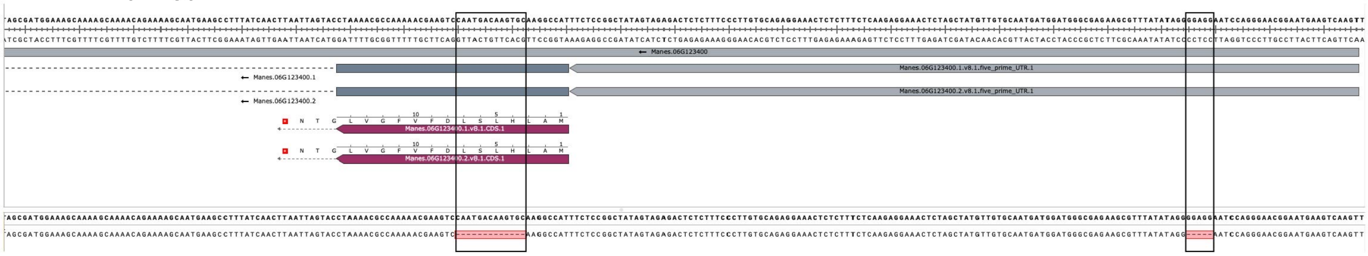

13 bp Deletion: CAATGACAAGTGC

5 bp Deletion: AGGGG

B.

KE269 haplotype 2

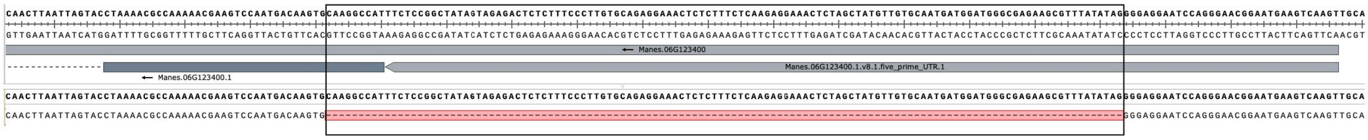

122 bp Deletion: CAAGGCCATTTCTCCGGCTATAGTAGAGACTCTCTTTCCCTTGTGCAGAGGAAAC TCTCTTTCTCAAGAGGAAACTCTAGCTATGTTGTGCAATGATGGATGGGCGAGAAGCGTTTATATAG

C.

Line 27 haplotype 1

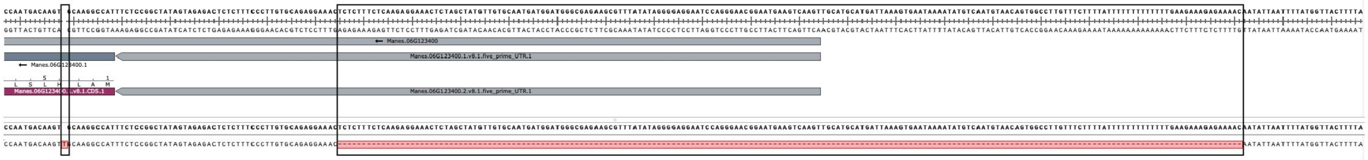

1 bp insertion: T

164 bp Deletion: TCTCTTTCTCAAGAGGAAACTCTAGCTATGTTGTGCAATGATGGATG GGCGAGAAGCGTTTATATAGGGGAGGAATCCAGGGAACGGAATGAAGTCAAGTTGC ATGCATGATTAAAGTGAATAAAATATGTCAATGTAACAGTGGCCTTGTTTCTTTTATTTT TTTTTTTTTGAAGAAAGAGAAAAC

D.

Line 30 haplotype 1

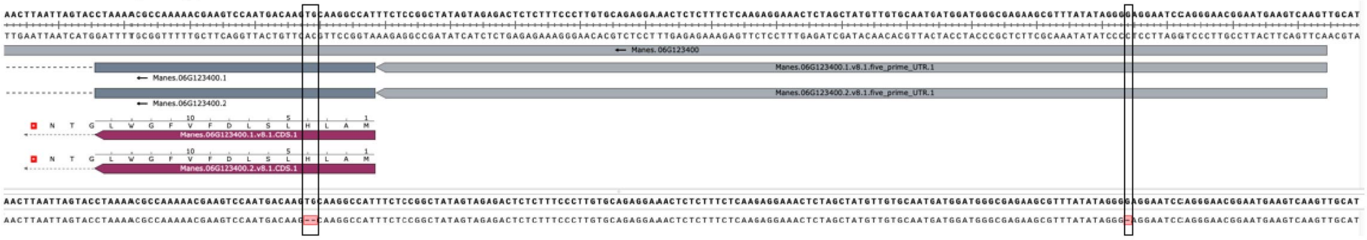

2 bp deletion: TG

1 bp deletion: G

Supplementary Figure S3: Whole genome sequencing to confirm MeSWEET10a mutant lines edits
Each diagram shows the genomic sequence followed by Manes.06G123400 annotation; below the annotation is the alignment of the de novo assembled contig. Differences between the genome and contig are in red, outlined by a box, and described below the box. **A)** Edits generated in Line KE338 on haplotype 1 (top) and haplotype 2 (bottom). **B)** Edits generated in line KE269 on haplotype 2. **C)** Edits generated in Line 27 on haplotype 1. Edits identified in line 27 using whole genome sequences appear to be a 164 bp deletion at EBE/TATA Box and 5'UTR rather than a 185 bp deletion which was identified by sanger-sequencing. **D)** Edits generated in Line 30 on haplotype 1. In lines 269, 27, and 30 only one clear mutant pattern was observed. These mutants are either homozygous or a second non-wildtype allele is present that was not able to be resolved but our genotyping methods.

A.

419-115 line 69B: Silent mutant — Construct 108

Potential 1 bp deletion upstream of TATA box and TAL20 EBE with 1bp deletion after start site.

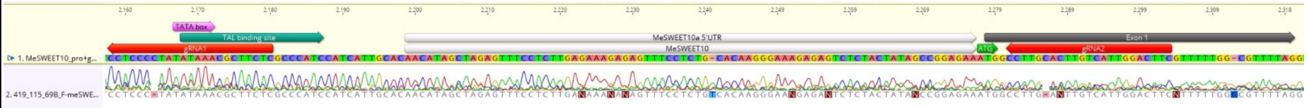

419-136 line 81: WT like transgenic or silent mutant — Construct 108

No edits detected

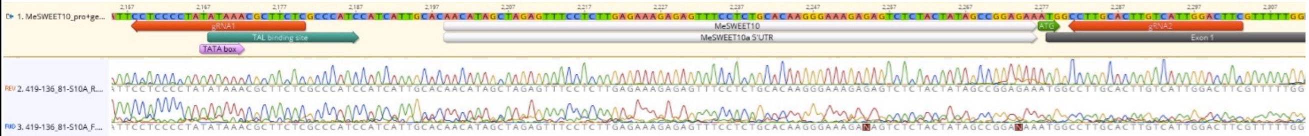

419-136 line 241*: TAL20 EBE mutant — Construct 108

Potential 16 bp deletion in TAL20 EBE with intact TATA box

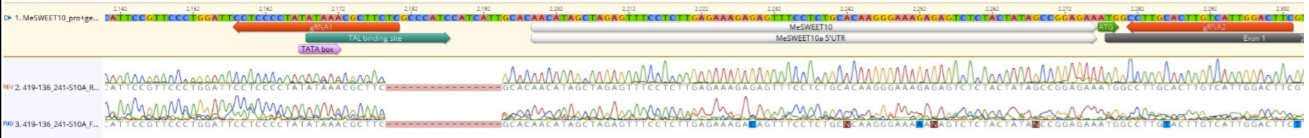

419-140 line 11: TATA box mutant, predicted non-functional — Construct 108

Potential 1 bp deletion in the TATA box.

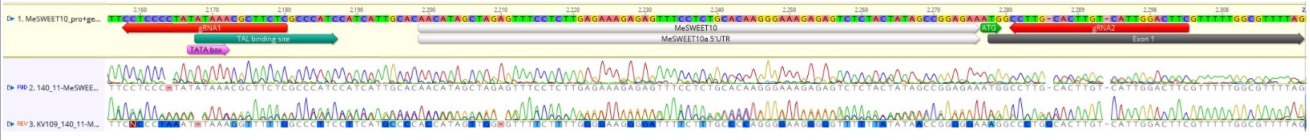

419-140 line 18: TATA box mutant, predicted non-functional — Construct 108

Potential 4bp deletion in TATA box upstream of TAL20 EBE and 6bp deletion downstream of ATG.

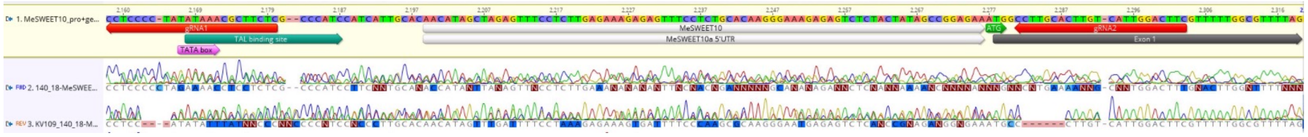

419-140 line 21: TATA box mutant, predicted non-functional — Construct 108

Potential 10bp deletion in TATA box upstream of TAL20 EBE.

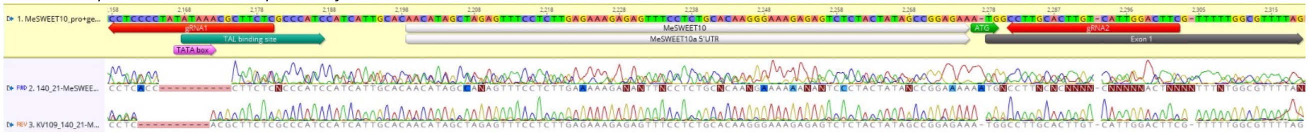

419-140 line 35: TATA box, TAL20 EBE, and 5'UTR mutant, predicted non-functional — Construct 108

Potential 76bp deletion including TATA box, TAL20 EBE, and 5'UTR

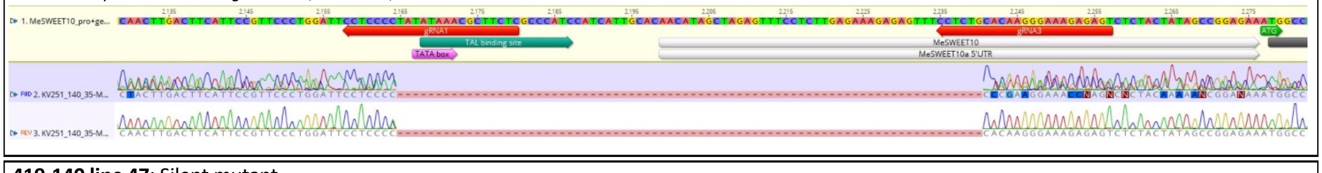

419-140 line 47: Silent mutant — Construct 108

2bp insertion upstream of TATA box and 1bp deletion in 5'UTR

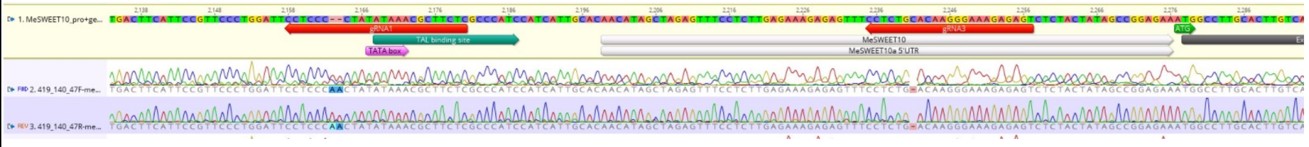

A.

419-140 line 49: TATA box and TAL20 EBE mutant — Construct 249

Potential 75bp deletion including TATA box, TAL20 EBE, and 5'UTR

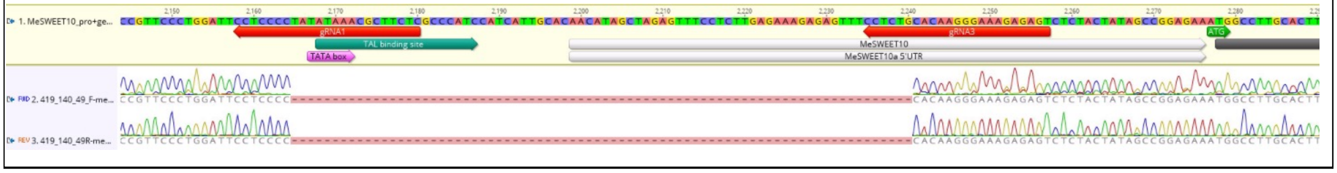

419-140 line 50: Silent mutant — Construct 249

Potential 1 bp deletion upstream of TATA box and 1bp insertion in 5'UTR

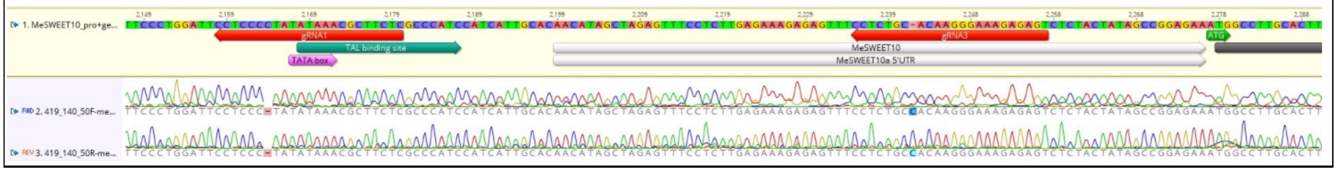

419-140 line 71: Silent mutant — Construct 250

Potential 1 bp deletion downstream of TATA box and possible deletions at gRNA 4 site

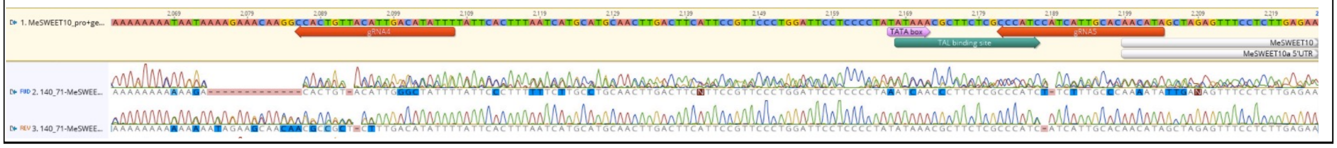

419-140 line 86: Silent mutant — Construct 250

Potential 2 bp deletion at gRNA 4 site and 4bp deletion downstream of the TATA box and TAL20 EBE site

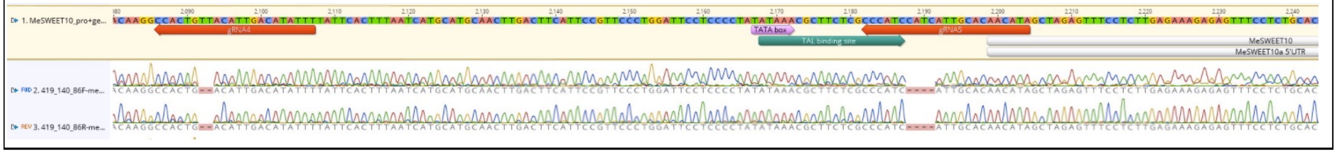

419-140 line 88: Silent mutant — Construct 250

Potential 2 bp deletion at gRNA 4 site and 4bp deletion downstream of the TATA box (Confirmed silent by RT-PCR)

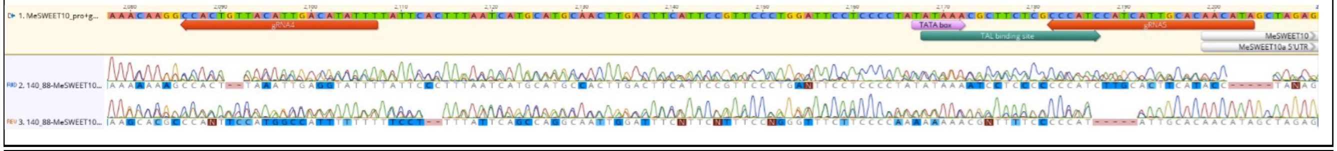

419-140 line 95: TATA Box and TAL20 EBE Mutant — Construct 250

Potential 96 bp deletion including TATA box, TAL20 EBE, and 5'UTR

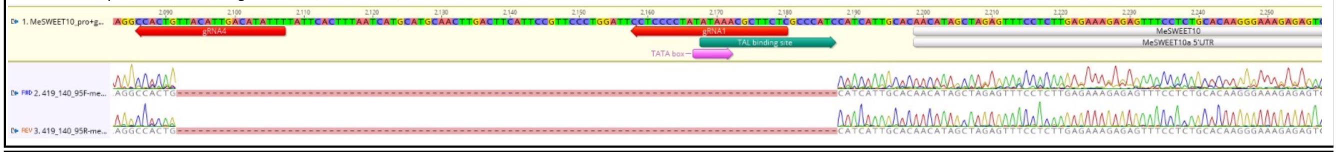

419-140 line 99: Silent Mutant

Potential 11 bp deletion at gRNA 4 site and 1 bp deletion downstream of TATA box and TAL20 EBE

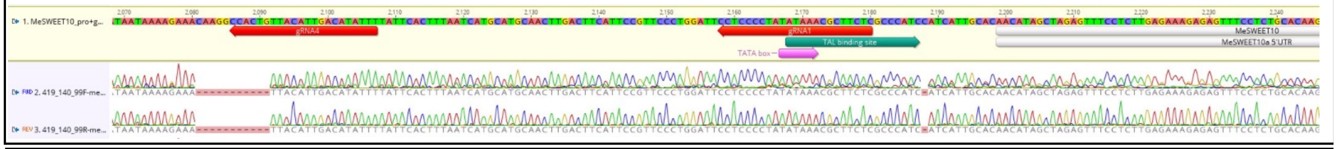

419-140 line 101: Silent Mutant

Potential 1 bp insertion at gRNA 4 site and 1 bp deletion downstream of TATA box and TAL20 EBE

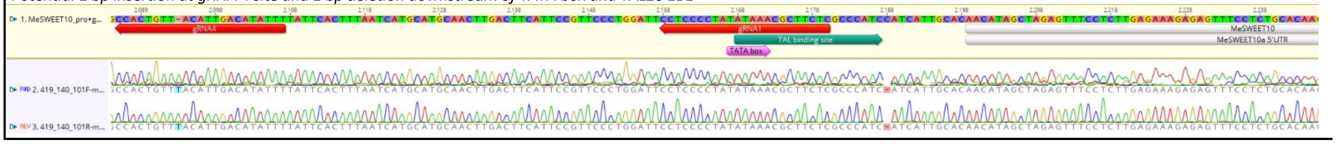

A.

419-140 line 108: Silent mutant

Construct 250

Possible 13bp deletion near gRNA4 and 1 bp deletion downstream of TATA box (Confirmed silent by RT-PCR)

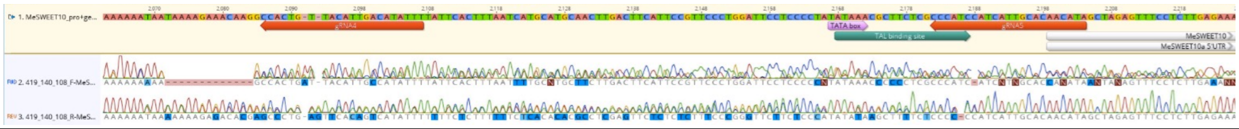

B.

419-140 line 27: Homozygous deletion and frameshift mutant

Construct 108

185 bp deletion including TATA box and TAL20 EBE, 1bp insertion after start site causing frameshift

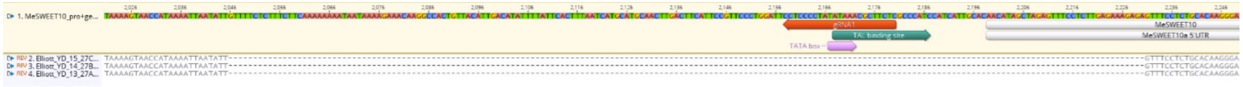

419-140 line 30: Homozygous frameshift mutant

Construct 108

1 bp insertion upstream of TATA box and TAL20 EBE, 2bp insertion after start site causing frameshift

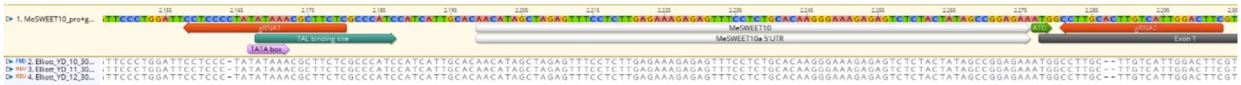

419-140 line 41: Biallelic deletion mutant

Construct 249

Allele 1: 5 bp deletion at TATA Box and 1 bp insertion in 5'UTR
Allele 2: 11 bp deletion including TATA box and TAL20 EBE and 1bp insertion in 5'UTR

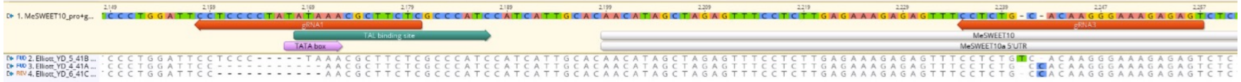

419-140 line 54: Biallelic deletion mutant

Construct 249

Allele 1: 5 bp deletion at TATA Box and TAL20 EBE site and 1 bp insertion in 5'UTR
Allele 2: 76 bp deletion including TATA box, TAL20 EBE and 5'UTR

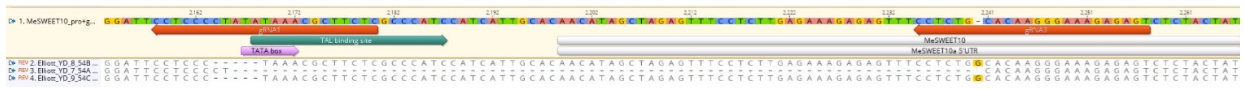

419-140 line 69A: Biallelic silent mutant

Construct 249

Allele 1: 1 bp insertion upstream of TATA Box and TAL20 EBE site and 1 bp deletion in 5'UTR
Allele 2: 1 bp deletion upstream of TATA box, and TAL20 EBE and and 1 bp insertion in 5'UTR

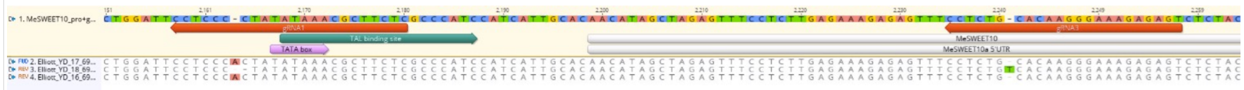

Supplementary Figure S4: Constructs 108, 249, and 250 Sanger-sequencing data from four replicate transformations **A)** Geneious screenshots showing Sanger-sequencing results for all lines obtained by additional transformation with constructs 108, 249, and 250. Mutation types and INDELs are described in text. '*' Denotes a line with EBE specific mutants that did not survive in tissue culture. **B)** Geneious screenshots of Oxford-Nanopore sequencing results of *E. coli* clones containing *MeSWEET10a* gDNA from lines 27, 30, 41, 54, and 69A plants.

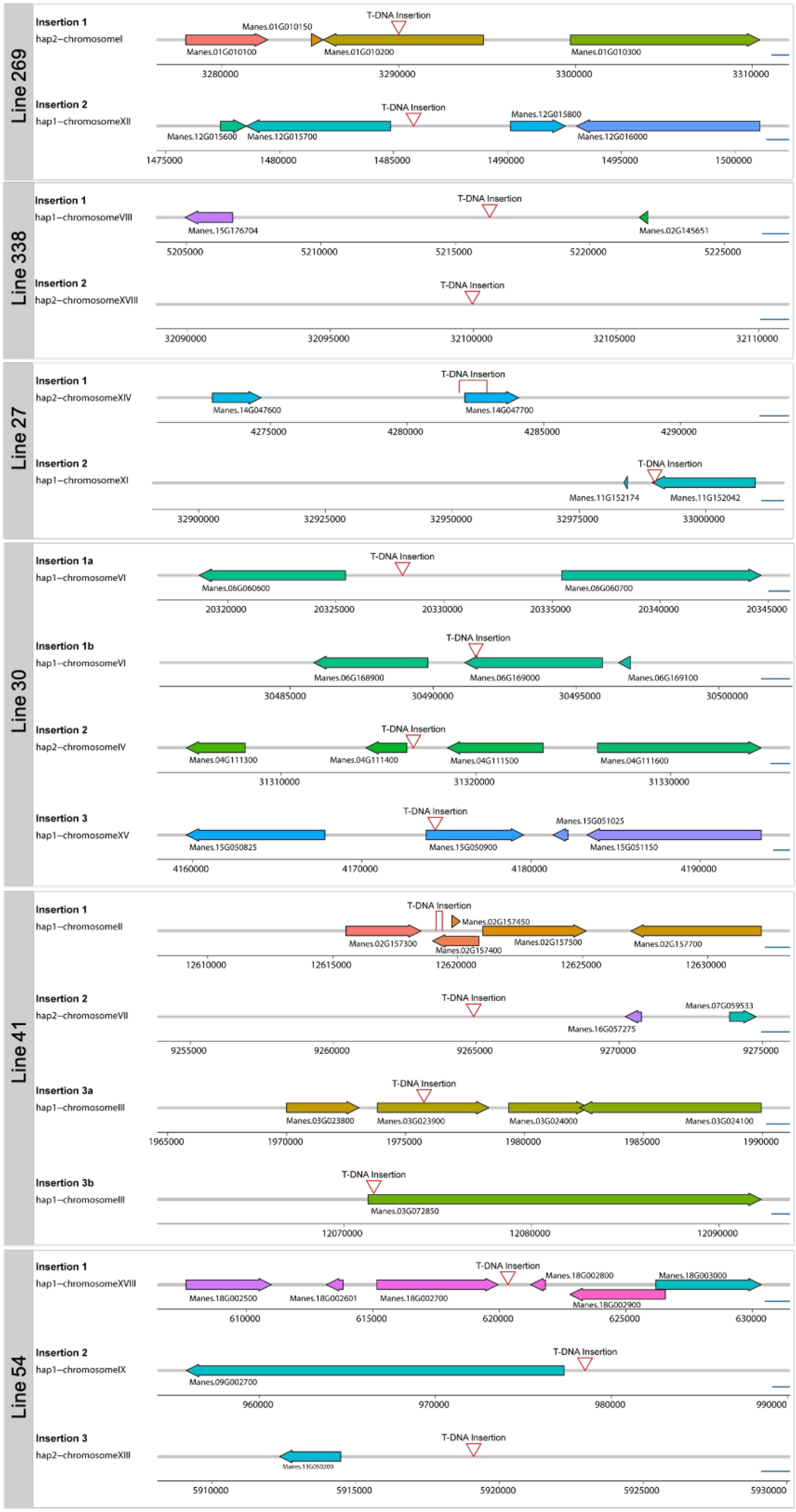

Supplementary Figure S5: Transgene insertion analysis

Graphic depicting transgene insertion number and location in lines 269, 338, 27, 30, 41, and 54. For each line, the total number of insertions is listed (left-hand side). For each insertion, the haplotype and chromosome location for the site of insertion is noted. Red triangles and brackets depict the site of transgene insertion. The bracket indicates a larger distance between insertion coordinates. Nearby genes are annotated with their Manes ID.

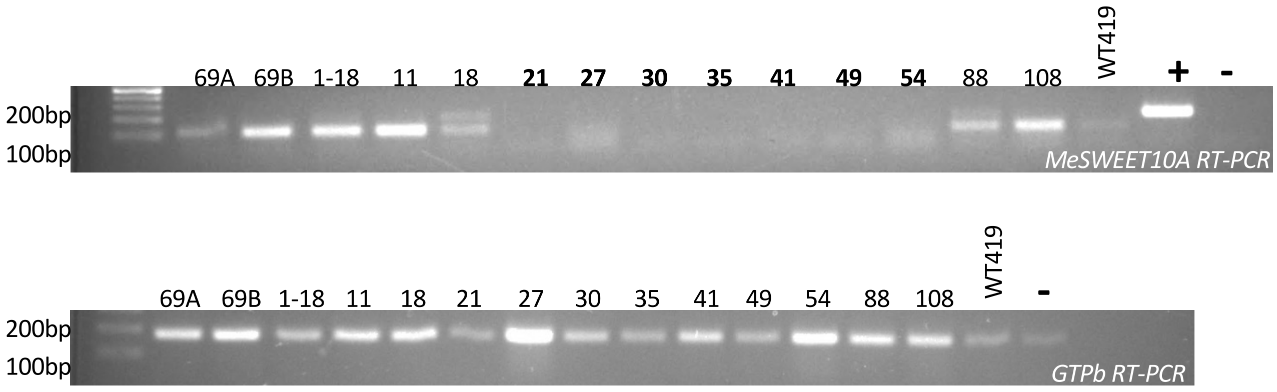

Supplementary Figure S6: Xpm infected detached leaf RT-PCR

RT-PCR of wildtype (WT419) cassava and *MeSWEET10a* mutant lines (69A, 69B, 1-18, 11, 18, 21, 27, 30, 35, 41, 49, 54, 88 and 108) detached leaves from plantlets infected with Xpm. The top gel shows results of RT-PCR with primers amplifying *MeSWEET10a* with an expected product size of 123 bp. The bottom gel shows results of RT-PCR with primers amplifying the housekeeping gene GTPB as a control for sample loading with an expected product size of 184 bp. '+' denotes a WT419 gDNA positive control. '-' denotes a negative water control. The bold text represents mutants that lack TAL20 mediated induction of *MeSWEET10a*.

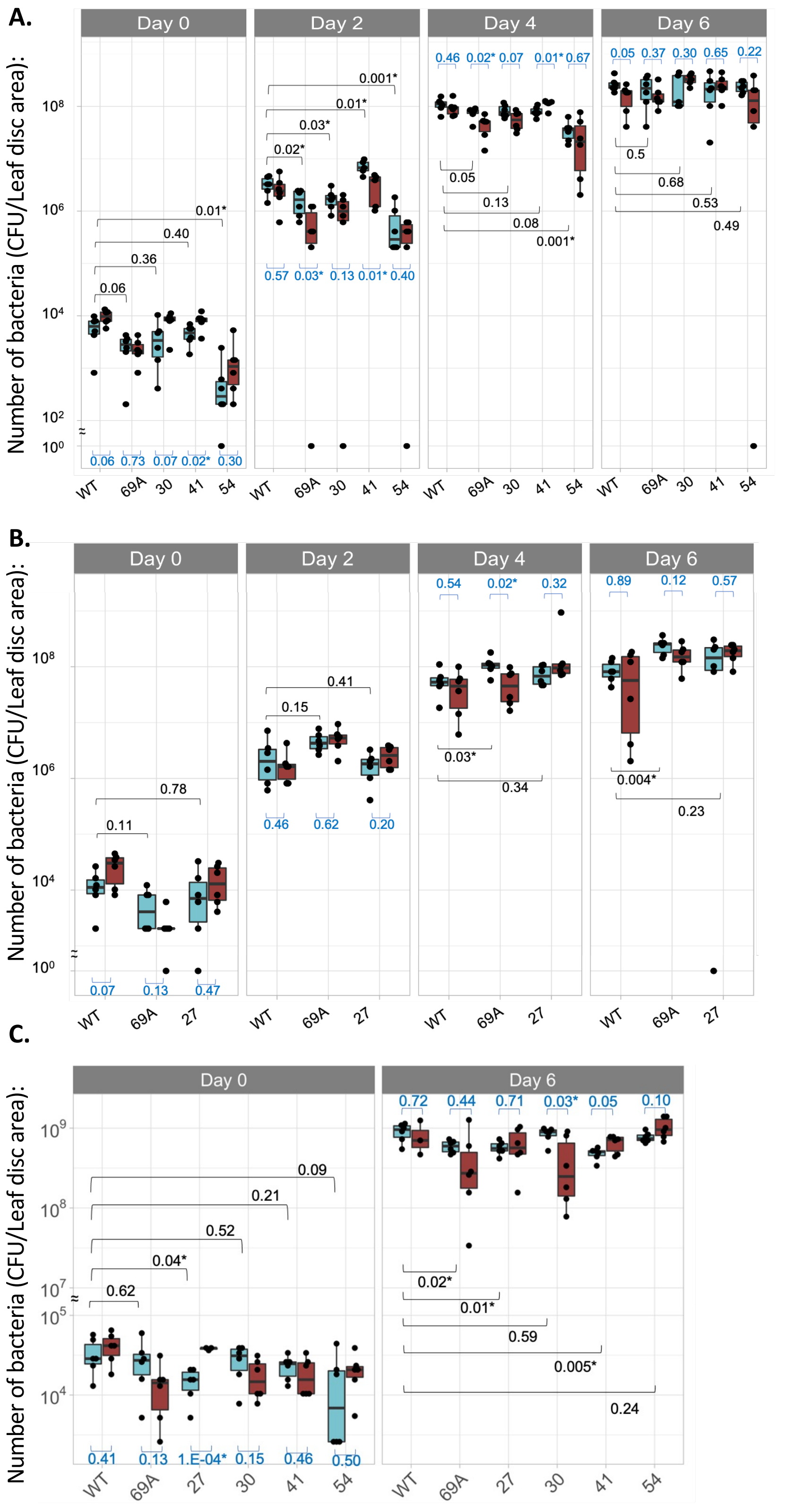

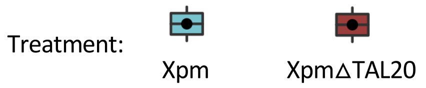

Supplementary Figure S7: Additional replicates of bacterial growth assays

A) Replicate 1 bacterial growth assay for WT, line 69A, 30, 41, and 54 plants. Number of bacteria in cassava leaves measured at 0, 2, 4, and 6DPI post syringe infiltration with Xpm (blue) and XpmΔTAL20 (red) treatments. **B)** Replicate 1 bacterial growth assay for mutant line 27. Along with WT and 69A controls. Number of bacteria in cassava leaves measured at 0, 2, 4, and 6DPI post syringe infiltration with Xpm (blue) and XpmΔTAL20 (red) treatments. **C)** Replicate 2 bacterial growth assay for WT, line 69A, 27, 30, 41, and 54 plants. Number of bacteria in cassava leaves measured at 0 (left) and 6DPI (right) post syringe infiltration with Xpm (blue) and XpmΔTAL20 (red) treatments. For all box plots, Colony Forming Units (CFU/cm2, Y-axis) are plotted by plant genotype (X-axis) tested (wildtype or mutant). Black dots represent technical replicates from one independent bacterial growth experiment. Results of statistical analyses (Unpaired student's t-test with unequal variance) comparing the difference between Xpm growth across wild-type and mutant cassava genotypes infected with Xpm. P-values are shown above or below brackets indicating the comparison types for statistical analyses. Black dots represent individual water-soaked lesions from three independent water-soaking assay experiments combined. In all boxplots, the calculated *p*-values (Unpaired Student's T-test with unequal variance) are shown above or below each box plot. Black text represents Xpm comparisons between WT and mutant infected plants. Blue text represents comparisons between Xpm and XpmΔTAL20 within each genotype. Dots outside whiskers represent outliers based on default settings of the R package ggplot2. The horizontal line within the box represents the median sample value. The ends of the boxes represent the 3rd (Q3) and 1st (Q1) quartiles. The whiskers show values that are 1.5 times interquartile range (1.5xIQR) above and below Q1 and Q3.

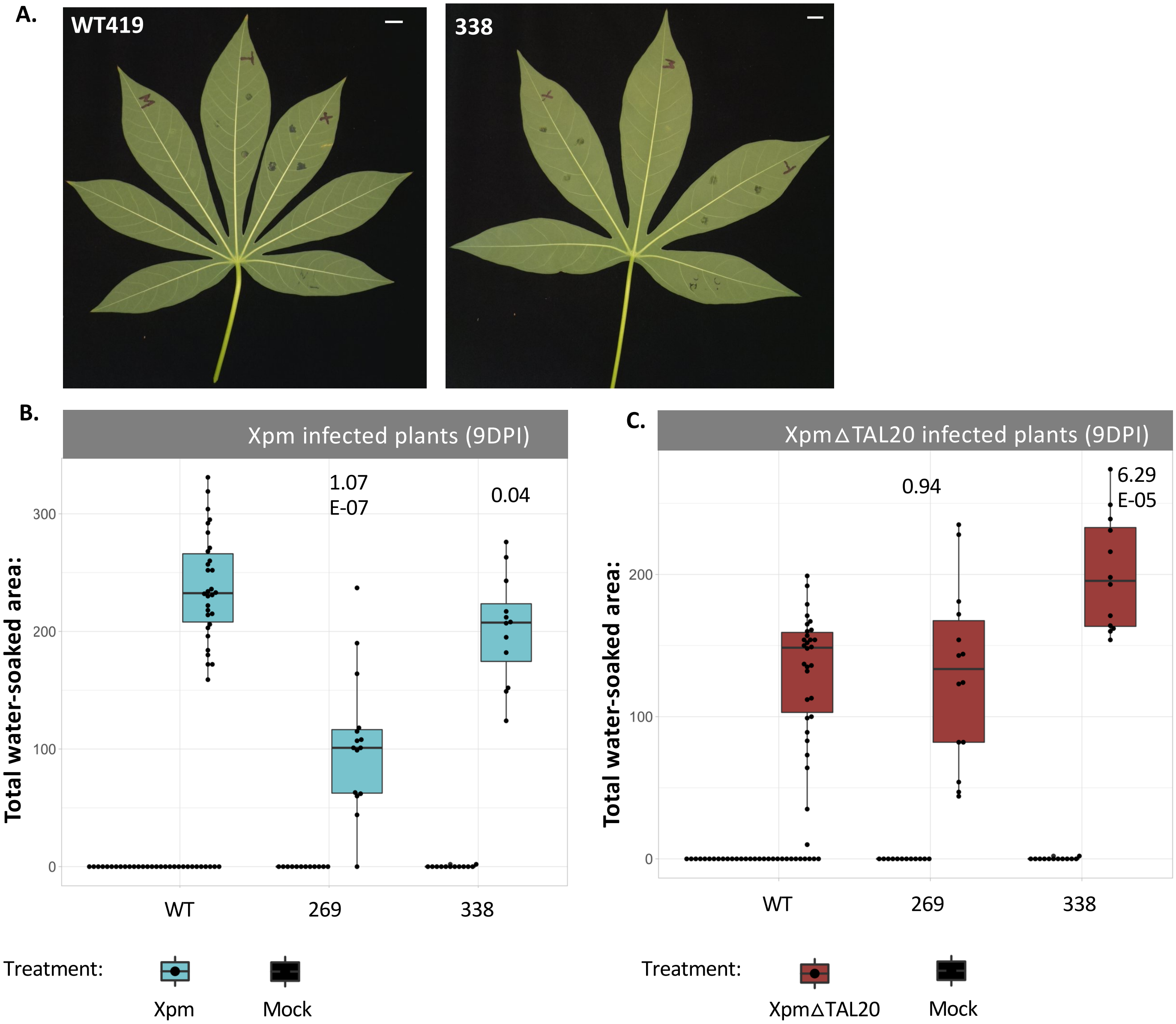

Supplementary Figure S8: Lines 269 and 338 water-soaked lesion assay

A) Representative images of infected wildtype (left) and mutant line 338 (right) cassava leaves detached from the plant and imaged at 6DPI. X= Xpm, T=XpmΔTAL20, and M=Mock. Scale bar = 1cm. B) Total water-soaked area (pixels, y-axis) of mock and Xpm infected plants (genotypes, x-axis) at 9DPI. C) Total water-soaked area (pixels, y-axis) of mock and XpmΔTAL20 infected plants (genotypes, x-axis) at 9DPI. Black dots represent individual water-soaked lesions from three independent water-soaking assay experiments combined. Calculated p-values (Unpaired Student's T-test with unequal variance) comparing mutant line to wildtype water-soaked area shown above each box plot. For all box plots, dots outside whiskers represent outliers. The horizontal line within the box represents the median sample value. The ends of the boxes represent the 3rd (Q3) and 1st (Q1) quartiles. The whiskers show values that are 1.5 times interquartile range (1.5xIQR) above and below Q1 and Q3.

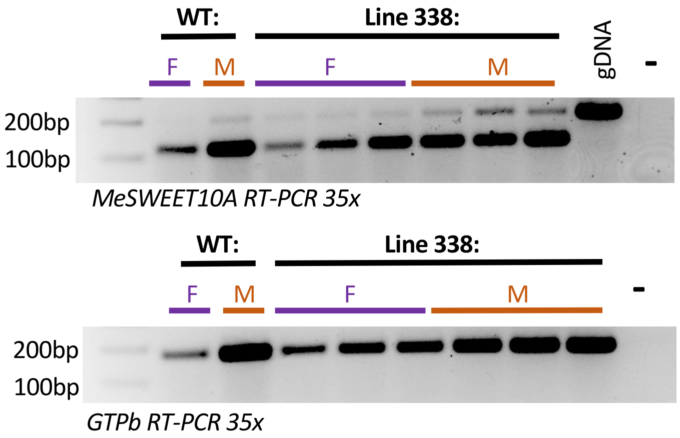

Supplementary Figure S9: RT-PCR of wildtype and line 338 flowers
RT-PCR 35x of WT419 (Left) and line 338 (Right) female (F, Purple) and male flowers (M, Orange) collected from field grown plants. The top gel shows results of RT-PCR with primers amplifying *MeSWEET10a* with an expected product size of 123 bp. Genomic DNA from WT419 leaf tissue is included as a positive control and '-' denotes a negative water control. The bottom gel shows results of RT-PCR with primers amplifying the housekeeping gene GTPB as a control for sample loading with an expected product size of 184 bp.

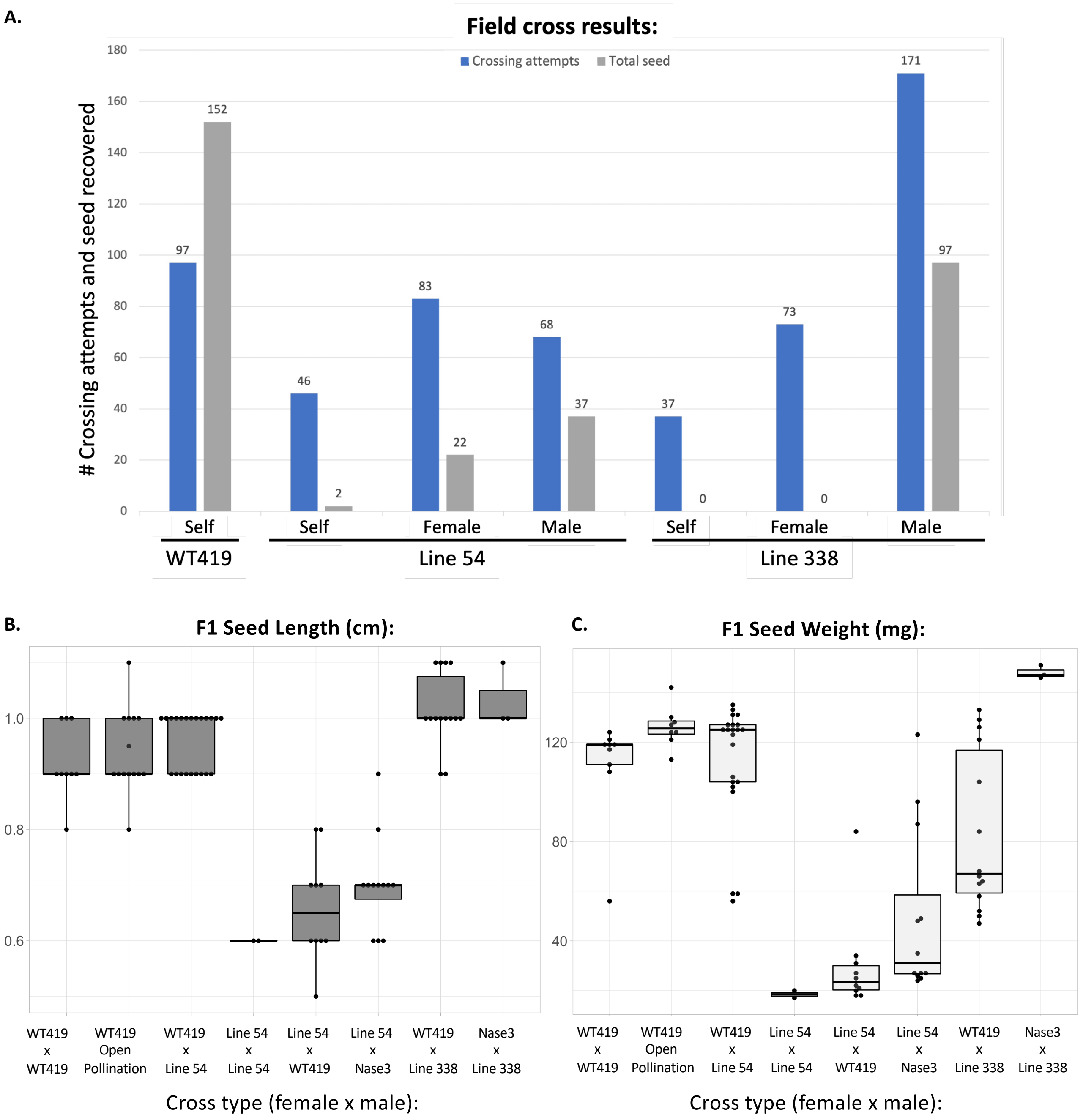

Supplementary Figure S10: *MeSWEET10a* mutant field cross results and F1 seed traits
A) Number of attempted crosses (blue) and seed recovered (grey) for WT419 self-crosses and all cross types with line 54 and line 338 female or male flowers. Measurements recorded for both **B)** **s**eed length (cm) and **C)** seed weight (mg) of F1 seed recovered from WT419 self-crosses, WT419 open pollinated crosses, all cross types with line 54 and line 338 female and male flowers. Dots outside whiskers represent outliers based on default settings of the R package ggplot2. The horizontal line within the box represents the median sample value. The ends of the boxes represent the 3rd (Q3) and 1st (Q1) quartiles. The whiskers show values that are 1.5 times interquartile range (1.5xIQR) above and below Q1 and Q3.

A.

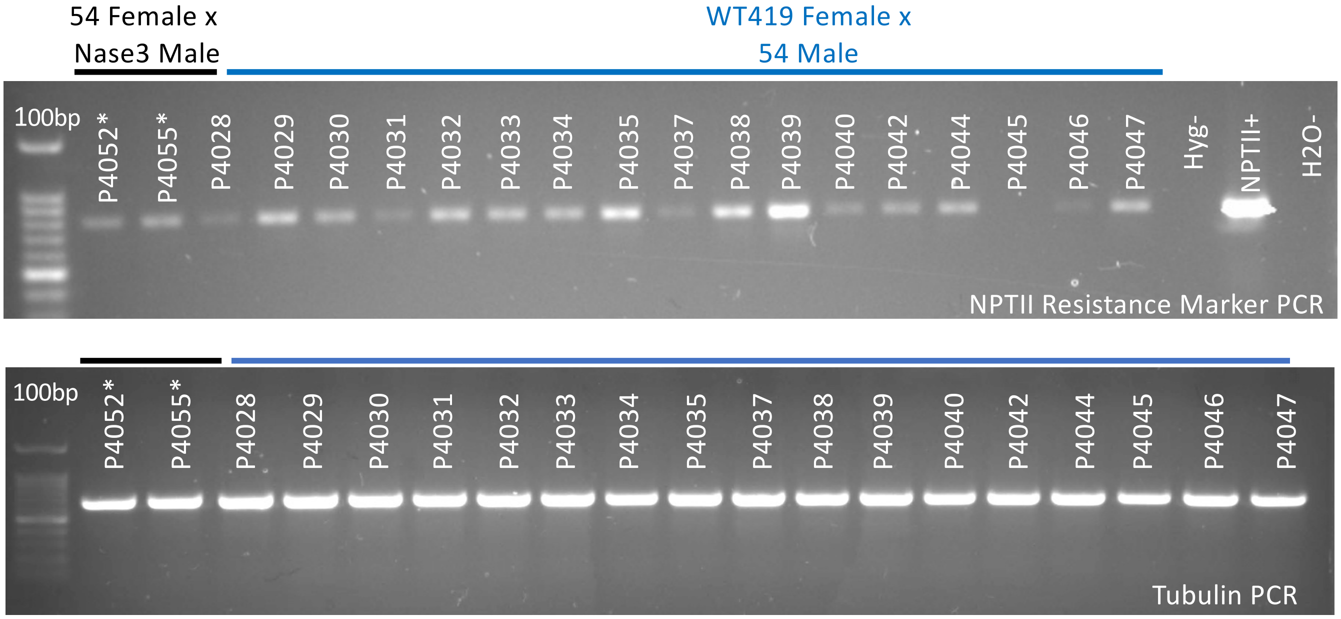

B.

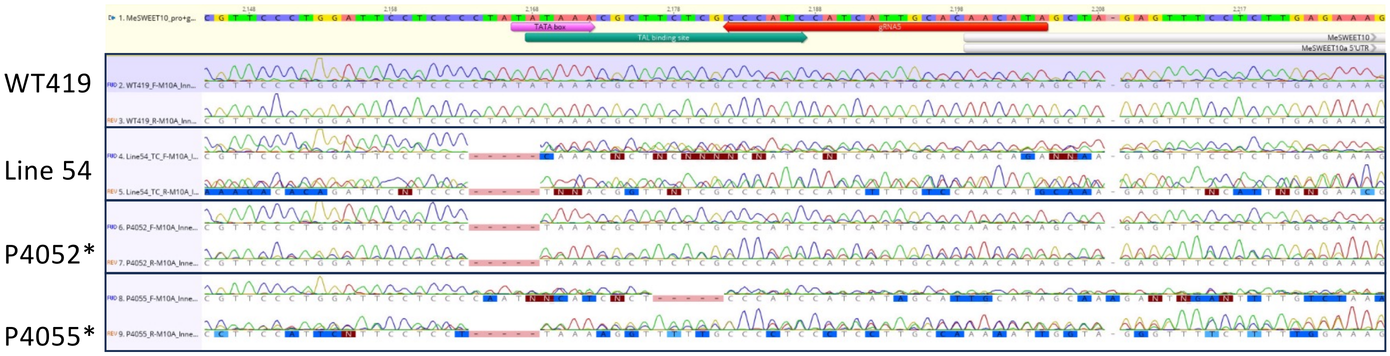

Supplementary Figure S11: Genotyping Line #54 *MeSWEET10a* mutant F1 progeny

A) Results from NPTII Resistance marker PCR (top gel, ~800 bp product) and Tublin PCR (bottom gel,~670 bp PCR, DNA loading control) on gDNA extracted from line #54 F1 progeny that successfully germinated. *denotes two seed that germinated from line #54 female x Nase3 male crosses. **B)** Geneious screenshots of Sanger-Sequencing completed on the two-line #54 female x Nase3 male derived seed with WT419 and Line #54 Sanger-sequencing included.

Supplementary Table S1: Transformation results

Construct:	Total settled cell volume (mL)[a]:	Callus selected (Stage 2):	Mature lines recovered (MS2):	Lines characterized:	Lines with confirmed edits:
108 (First generation)	2900	344	30	5	4
108	11800	206	7	7	6
249	14250	264	8	8	8
250	13300	324	9	9	9

Footnote: [a] The total settled cell volume (SCV) of callus used for each construct was calculated by taking the sum of SCV measured for each individual construct replicates across all transformations.

Supplementary Table S2: Plant morphology measurements

Plant background:	WT419:	Line #27:	Line #30:	Line #41:	Line #54:	Line #69A:
Average Plant height (cm)	44.87 ±1.44	47.15 ±3.18	54.25 ±3.44	46.89 ±3.42	41.70 ±1.32	46.11 ±7.31
T.test (WT vs Line)		0.35	0.03	0.42	0.05	0.85
Average number of nodes	34.33 ±0.58	32.33 ±3.21	34.00 ±2.31	30.33 ±2.08	35.67 ±0.58	31.00 ±1.41
T.test (WT vs Line)		0.39	0.34	0.07	0.05	0.16
Average Internode length (above the woody transition) (cm)	0.42 ±0.09	0.53 ±0.09	0.40 ±0.08	0.74 ±0.17	0.56 ±0.08	1.00 ±0.29
T.test (WT vs Line)		0.23	0.72	0.06	0.13	0.20
Average Internode length (below the woody transition) (cm)	0.26 ±0.09	0.45 ±0.17	0.24 ±0.00	0.56 ±0.14	0.45 ±0.12	0.75 ±0.06
T.test (WT vs Line)		0.18	0.67	0.05	0.11	0.01
Leaf lobe # average	6.78 ±0.67	6.11 ±0.93	6.00 ±0.71	6.89 ±0.33	7.00 ±0.00	5.33 ±0.52
T.test (WT vs Line)		0.08	0.07	0.69	0.42	0.01
Petiole length average (cm)	13.86 ±2.67	15.45 ±1.71	15.79 ±3.30	13.67 ±2.71	13.40 ±1.88	14.87 ±0.74
T.test (WT vs Line)		0.44	0.51	0.94	0.81	0.57
Central lobe length: 3 representatives per plant average (cm)	14.18 ±0.94	15.38 ±1.14	17.03 ±1.65	15.41 ±1.12	12.87 ±0.83	13.20 ±0.64
T.test (WT vs Line)		0.02	0.03	0.19	0.03	0.06
Central lobe width: 3 representatives per plant Average (cm)	3.62 ±0.24	4.04 ±0.25	4.10 ±0.39	4.42 ±0.38	3.31 ±0.23	3.57 ±0.28
T.test (WT vs Line)		0.05	0.03	0.04	0.03	0.75
Whole leaf width: 3 representatives per plant average (cm)	22.08 ±2.23	23.42 ±1.77	24.41 ±2.88	23.14 ±2.12	20.03 ±1.90	17.61 ±0.45
T.test (WT vs Line)		0.31	0.13	0.57	0.16	0.04

Mean values are displayed +/- SD. A minimum of three replicate samples were included except for line 69A which had 2 replicates.

Seed ID	Date of Cross	Female	Male	Seed length (cm)	Seed width (cm)	Weight (mg)	Sink(s) Float(f)	Germinated
P4006	6/21/23	TME419	54	0.9	0.5	126.7	sink	Yes
P4007	7/17/23	54	NASE3	0.6	0.4	23.6	float	No
P4008	7/17/23	54	NASE3	0.7	0.3	27.2	float	No
P4009	7/17/23	54	NASE3	0.7	0.35	24.5	sink	No
P4010	7/17/23	54	NASE3	0.7	0.4	47.8	float	No
P4011	6/21/23	TME419	TME419	0.9	0.5	123.7	sink	Yes
P4012	6/21/23	TME419	TME419	0.9	0.5	111.2	sink	Yes
P4013	6/21/23	TME419	TME419	1	0.5	119.2	float	Yes
P4014	6/21/23	TME419	TME419	0.9	0.5	119.4	sink	Yes
P4015	6/21/23	TME419	TME419	0.9	0.5	118.7	sink	Yes
P4016	5/30/23	NASE3	338	1	0.6	150.9	sink	Yes
P4017	5/30/23	NASE3	338	1.1	0.6	146.1	sink	Yes
P4018	5/30/23	NASE3	338	1	0.6	146.8	float	No
P4028	8/2/23	TME419	54	0.9	0.4	104	sink	Yes
P4029	8/2/23	TME419	54	0.9	0.5	100.1	sink	Yes
P4030	8/2/23	TME419	54	0.9	0.5	101.6	sink	Yes
P4031	8/2/23	TME419	54	1	0.5	130.5	sink	Yes
P4032	8/2/23	TME419	54	1	0.5	134.8	sink	Yes
P4033	8/2/23	TME419	54	1	0.5	124.7	float	Yes
P4034	8/2/23	TME419	54	1	0.5	124.5	sink	Yes
P4035	8/2/23	TME419	54	1	0.5	124.8	sink	Yes
P4036	8/2/23	TME419	54	0.9	0.5	56.1	float	No
P4037	8/2/23	TME419	54	0.9	0.5	124.9	sink	Yes
P4038	8/2/23	TME419	54	0.9	0.5	125.4	float	Yes
P4039	8/2/23	TME419	54	1	0.5	133.4	sink	Yes
P4040	8/2/23	TME419	54	1	0.5	119.4	sink	Yes
P4041	8/2/23	TME419	54	1	0.5	59.2	float	No
P4042	8/2/23	TME419	54	1	0.5	130.6	sink	Yes
P4043	8/2/23	TME419	54	0.9	0.5	59.2	float	No
P4044	8/2/23	TME419	54	1	0.5	127.4	sink	Yes
P4045	8/2/23	TME419	54	0.9	0.5	106.3	float	Yes
P4046	6/27/23	TME419	54	1	0.5	127.3	sink	Yes
P4047	6/27/23	TME419	54	1	0.5	104.4	float	Yes
P4048	6/27/23	TME419	54	1	0.5	122.8	sink	Yes
P4049	8/17/23	54	NASE3	0.6	0.3	26.5	float	No
P4050	8/17/23	54	NASE3	0.6	0.4	34.9	float	No
P4051	8/17/23	54	NASE3	0.7	0.5	96.1	float	No
P4052	7/17/23	54	NASE3	0.7	0.4	86.9	float	Yes
P4053	7/17/23	54	NASE3	0.7	0.3	26.2	float	No
P4054	7/17/23	54	NASE3	0.7	0.3	27.1	float	No
P4055	7/16/23	54	NASE3	0.9	0.5	122.7	sink	Yes
P4056	7/16/23	54	NASE3	0.8	0.5	48.8	float	No
P4145	6/21/23	TME419	TME419	0.8	0.5	121.1	sink	Yes
P4146	6/21/23	TME419	TME419	1	0.5	55.6	float	No
P4147	6/21/23	TME419	TME419	0.9	0.4	108.2	sink	Yes
P4148	6/21/23	TME419	TME419	1	0.5	116.9	sink	Yes
P4149	9/18/23	54	TME419	0.6	0.4	17.6	float	No
P4150	9/18/23	54	TME419	0.5	0.4	17.7	float	No
P4151	9/18/23	54	TME419	0.8	0.4	34	float	No
P4152	8/15/23	54	TME419	0.6	0.4	19.9	float	No
P4153	8/15/23	54	TME419	0.6	0.4	20.9	float	No
P4154	8/15/23	54	TME419	0.7	0.5	84.1	float	Yes
P4155	9/18/23	54	TME419	0.7	0.4	30.6	float	No
P4156	9/18/23	54	TME419	0.6	0.4	25.2	float	No
P4157	9/18/23	54	TME419	0.7	0.3	21.6	float	No
P4158	9/18/23	54	TME419	0.8	0.5	26.5	float	No
P4159	8/22/23	54	54	0.6	0.4	19.8	float	No
P4160	8/22/23	54	54	0.6	0.4	17.1	float	No
P3760	10/10/22	TME419	338	1	0.6	63.6	float	No
P3761	10/10/22	TME419	338	0.9	0.5	84.1	float	Yes
P3788	10/13/22	TME419	338	1.1	0.5	63	float	No
P3789	10/13/22	TME419	338	1.1	0.5	57.5	float	No
P3790	10/13/22	TME419	338	1.1	0.5	103.9	float	Yes
P3791	10/13/22	TME419	338	1	0.5	52.2	float	No
P3792	10/13/22	TME419	338	1	0.5	46.7	float	No
P3793	10/13/22	TME419	338	1	0.5	121.1	sink	Yes
P3794	10/13/22	TME419	338	1.1	0.5	133.1	sink	Yes
P3795	10/13/22	TME419	338	1	0.5	50.1	float	No
P3825	10/14/22	TME419	338	1	0.5	126.2	sink	Yes
P3826	10/14/22	TME419	338	1	1	66	float	No
P3972	10/14/22	TME419	338	0.9	0.5	67.6	float	No
P3973	10/14/22	TME419	338	1	0.5	128.8	sink	Yes
P3974	Spring 2022	TME419	open pollination	1.1	0.6	141.9	sink	Yes
P3975	Spring 2022	TME419	open pollination	0.9	0.5	121.1	sink	Yes
P3976	Spring 2022	TME419	open pollination	0.9	0.5	124.3	sink	Yes
P3977	Spring 2022	TME419	open pollination	0.9	0.5	126.5	sink	Yes
P3978	Spring 2022	TME419	open pollination	0.95	0.5	127.8	sink	Yes
P3979	Spring 2022	TME419	open pollination	1	0.5	113.2	sink	Yes
P3980	Spring 2022	TME419	open pollination	0.8	0.5	124.4	sink	Yes
P3981	Spring 2022	TME419	open pollination	1	0.6	129.6	sink	Yes
P3711	Spring 2022	TME419	open pollination	1.0	0.6	Not recorded	float	No
P3712	Spring 2022	TME419	open pollination	0.9	0.6	Not recorded	sink	Yes
P3723	Spring 2022	TME419	open pollination	1.0	0.6	Not recorded	sink	Yes
P3724	Spring 2022	TME419	open pollination	0.9	0.6	Not recorded	float	Yes
P3736	Spring 2022	TME419	open pollination	0.9	0.6	Not recorded	sink	Yes
P3737	Spring 2022	TME419	open pollination	0.9	0.5	Not recorded	float	No

Supplementary Table S3: Seed measurements and germination
Table of recorded information for F1 seed recovered from the field and tested for germination. Seed ID, Date of Cross, Female, Male, Seed length (cm), width (cm), weight (mg), Float test results (sink or float), and germination data (yes or no) are reported.

Primer Number:	Sequence: (5'-3')	Description:
MeSWEET10a outer F (150)	GGGAATGAGAGTGTGTGGTTAG	Genotyping primer avoiding EBE repair
MeSWEET10a outer R (151)	GCAGTCAAGGTGAGGAAGAAT	Genotyping primer EBE repair
MeSWEET10a inner F (21)	GTACCCTTCCTCAACACCC	Genotyping primer
MeSWEET10a inner R (22)	GCTGCAAAACCATCGAGCAA	Genotyping primer
MeSWEET10a RT-PCR F (200)	CTTACTGTGTACCTTATCTATGCCACAAAGAAG	RT-PCR *MeSWEET10a* expression primer
MeSWEET10a RT-PCR R (201)	GCCATGTGTAAGGAAAAGAGTTAAGATAGCG	RT-PCR *MeSWEET10a* expression primer
Actin RT-PCR F (37)	ACAGTGTCTGGATCGGAGGATC	RT-PCR Actin housekeeping gene expression primer
Actin RT-PCR R (38)	GAAGCACTTCCTGTGGACGATG	RT-PCR Actin housekeeping gene expression primer
GTPb RT-PCR F (109)	CCTCAAAGGCTGAGCCACAGA	RT-PCR GTPb housekeeping gene expression primer
GTPb RT-PCR R (110)	GGGAGAAACAATACAGGCACCAATCAC	RT-PCR GTPb housekeeping gene expression primer
A_CmYLCV-F (113)	TGCTCTTCGCGCTGGCAGACATACTGTCCCAC	CmYLCV primer for CRISPR/Cas9 construct assembly
B_10gRNA1-R (114)	TCGTCTCCTAAACGCTTCTCCTGCCTATACGGCAGTG AACCTG	gRNA1 R primer for CRISPR/Cas9 construct assembly
C_10gRNA1-F (115)	TCGTCTCATTTATATAGGGGGTTTTAGAGCTAGAAAT[illegible]	gRNA1 F primer for CRISPR/Cas9 construct assembly
D_10gRNA2-R (116)	TCGTCTCATCATTGGACTTCCTGCCTATACGGCAGTGA AC	gRNA2 R primer for CRISPR/Cas9 construct assembly
C_10gRNA2-F (117)	TCGTCTCAATGACAAGTGCAGTTTTAGAGCTAGAAAT AGC	gRNA2 F primer for CRISPR/Cas9 construct assembly
E_35Sterm-R (118)	TGCTCTTCTGACCTGCCTATACGGCAGTGAAC	35S terminator primer for CRISPR/Cas9 construct assembly
B_10gRNA4-R (236)	TCGTCTCCTTGACATATTTTCTGCCTATACGGCAGTGA ACCTG	gRNA4 R primer for CRISPR/Cas9 construct assembly
C_10gRNA4-F (237)	TCGTCTCATCAATGTAACAGGTTTTAGAGCTAGAAAT AGC	gRNA4 F primer for CRISPR/Cas9 construct assembly
D_10gRNA5-R (238)	TCGTCTCATTGCACAACATACTGCCTATACGGCAGTG AAC	gRNA5 R primer for CRISPR/Cas9 construct assembly
C_10gRNA5-F (239)	TCGTCTCAGCAATGATGGATGTTTTAGAGCTAGAA ATAGC	gRNA5 F primer for CRISPR/Cas9 construct assembly
Tubulin F (95)	GAT CCTACTGGGAAGTACATTGG-3	Housekeeping DNA loading control primer
Tubulin R (96)	CTGCATTCTCCACCAACTGA	Housekeeping DNA loading control primer
NPTII F (93)	ATGATTGAACAAGATGGATTGCAC	Primer to amplify NPTII resistance cassette in T-DNA
NPTII R (94)	TCAGAAGAACTCGTCAAGAAGGCG-	Primer to amplify NPTII resistance cassette in T-DNA
HYG F (101)	TAGCGAGAGCCTGACCTATT	Primer to amplify HYG resistance cassette in T-DNA (used as a negative control)
HYG R (102)	GATGTTGGCGACCTCGTATT	Primer to amplify HYG resistance cassette in T-DNA (used as a negative control)

Supplementary Table S4: Primer list
Table of all primers used in this study. The primer name/stock number (left), sequence (middle), and description (right) are provided.
