# Supplementary material for: CRISPR/Cas9-generated mutations in a sugar transporter gene reduce cassava susceptibility to bacterial blight
Source: Plant Physiol. 2024 May 3;195(4):2566–78. doi: 10.1093/plphys/kiae243 (PMC11288762; doi:10.1093/plphys/kiae243)
Supplement: kiae243_Supplementary_Data [file pp.00918.2023-s03.pdf › pp.00918.2023-s03.pdf]

Plant Physiology®

CRISPR/Cas9 generated MeSWEET10a mutants show reduced susceptibility to cassava bacterial blight and produce viable seed

Kiona Elliott, Kira Veley, Greg Jensen, Kerrigan Gilbert, Joanna Norton, Lukas Kambic, Marisa Yoder, Alex Weil, Sharon Motomura-Wages, and Rebecca Bart

Review Timeline:

Submission Date:	21-Jun-2023
Editorial Decision:	06-Aug-2023
Revision Received:	30-Jan-2024
Editorial Decision:	22-Feb-2024
Revision Received:	02-Apr-2024
Accepted:	02-Apr-2024

06-Aug-2023

Dr. Rebecca Bart
Donald Danforth Plant Science Center
Bart Lab
St. Louis, Missouri

RE: CRISPR/Cas9 generated MeSWEET10a mutants show reduced susceptibility to cassava bacterial blight and produce viable seed

Dear Dr. Bart:

We have received reviews of your manuscript, which you may view at the end of this letter. On the basis of the advice received and my own evaluation, I believe that, pending revision, this article may ultimately be acceptable for publication in Plant Physiology.

Providing a more detailed description of the generation and molecular characterization of the edit lines is especially relevant. Both reviewers (and I agree with them) raised concerns about the molecular description of the edited lines, not just in terms of generated mutations but also expression in flowers and resulting phenotypes. Furthermore, the apparent similarity in bacterial titers between the wild-type and edited lines must be addressed. The difference in susceptibility seems primarily attributed to the water-soaked area.

When you are ready to submit your revision, click the below link and find the red arrow under your Author Tasks heading for this revision submission.

If you want to visit this version of the manuscript, you may click the link below and then click on the folder that reads "Post Decision Manuscripts".

Link Not Available

When uploading the revised version of this article, please be sure to include as complete an answer as possible to the referees' criticisms during the first round of reviews in the "response to reviewers" section.

Plant Physiology considers a revision for up to 60 days after a manuscript has been provisionally accepted; limited extension of this timeframe is at the discretion of the Monitoring Editor. A reminder notice will be sent to you 2 weeks before the deadline, at which time you may choose to contact the editorial office to request an extension; if so, please provide a justification for your request. After the expiration of this deadline, the article would need to be submitted as a resubmission and would be handled as such.

Thank you very much for giving us an opportunity to review this work. I look forward to receiving the next version.

Sincerely,

Board of Editors Plant Physiology

Want to add this revision deadline to your calendar? Click below!

-- Reviewer comments:
Reviewer #1 (Comments for the Author):

Elliott et al. generated MeSWEET10a cassava mutants using CRISPR/Cas9, and claim that these show a reduced susceptibility to cassava bacterial blight. This is an important result but the manuscript has multiple weaknesses, many minor but some more substantial.

1. Abstract: "CBB" is an undeclared abbreviation.
2. Results: It is unclear how the potential TAL20 effector binding site upstream of the MeSWEET10a start codon was identified. They could explain better why they conclude this is a genuine EBE
3. Several combinations of Cas9 guides were used, but there was little explanation of how these guides were selected or what exactly the intended editing outcome is. Were they trying to obtain small indels, a large indel between the guides, a targeted KI or all of these? How were the first pair of guides chosen, followed by additional guides later in the manuscript?
4. Transformation efficiency seems to have been variable, but it was not clearly reported. Can you report a transformation efficiency and how this was calculated?
5. Little effort was made to interpret the mutations' effects on the expression of the gene. It seems very likely that most of the lines will be knock-outs due to the deletion of the start codon, TATA box, or both, but RT-PCR data are required to assess the impact of the mutations. At a minimum, since the authors report that this gene is normally expressed in flowers, they should assess if the mutant alleles they generated are expressed in flowers.
6. It is not clear how they determined the lines' zygosity for the edits. What exactly is meant by "clone-seq"?
7. They claim to have recovered several T0 plants homozygous for the same NHEJ/MMEJ-induced indels. As these are messy processes, it is surprising that the recovered plants carry two haplotypes with the exact same mutations. Could they be missing alternative haplotypes in the lines they've genotyped? Have they confirmed the lines' inferred zygosity through crossing? I recognize cassava is more difficult to work with than Arabidopsis or tomato, but if they don't know because of technical difficulties, they should make this more clear.
8. When they sent samples for Sanger sequencing after digestion, did they also send WT samples? This could have nicely demonstrated that the observed mutations are genuine edits and not just artifacts of the methodology.
9. When the ability of Xanthomonas to induce the expression of MeSWEET10a was tested in Fig. 1E and Fig. 2F, there was no control for whether the edited lines can still express the gene at all, or if they were knocked-out. Flower tissue from the plants could have provided a nice positive control.
10. In Fig. 1E and Fig. 2F, you use different Xanthomonas strains. Why?
11. In Fig. 1E and Fig. 2F, you use different housekeeping genes as controls. Why?
12. In Fig. 2 A&B, guides 1, 2, and 3, but not guide 4, are shown. Why? Were no plants sourced from construct 250 genotyped?
13. "These results support the hypothesis that edits in these lines block induction of MeSWEET10a by TAL20." Here you interpreted the RT-PCR data as a failure of the pathogen to control the host's metabolism, but the experimental data provided does not bear this out. You may have abolished expression of the MeSWEET10a gene anywhere in the plant.
14. In Figure 3, how were the statistics done?
15. When challenged with the Xanthomonas phaseoli pv. manihotis pathogen, the edited lines did not harbour a reduced bacterial population after infiltration. The authors seem to disregard this negative result, and instead accept a reduction in the leaf water-soaked area as a truer sign of reduced disease susceptibility. This is not very convincing and should be more objectively discussed
16. "We conclude that MeSWEET10a mutant plants have decreased susceptibility to cassava bacterial blight." The current manuscript does not support such a bold claim, since bacterial titres appear unaltered.
17. "line #338 had been established in a field site outside Hilo, Hawaii along with WT419 plants. WT419 female and male flower buds were collected for RNA extraction and RT-PCR. Expression of MeSWEET10a was confirmed for both flower types" It is surprising that the authors tested the expression of MeSWEET10a in the WT line but did not test in the mutant.
18. Discussion: "The ideal MeSWEET10a mutant line, for CBB resistance, would contain edits at the TAL20 EBE site while maintaining an intact TATA box and gene coding sequence for native plant function." This sentence needs to be moved to the early Results/late Introduction.
19. "It is also possible that other TAL20 carrying Xpm strains may have effectors with redundant functions and therefore may not exhibit reduced virulence after infection in MeSWEET10a mutants." Is Xpm △TAL20 less virulent?

Reviewer #2 (Comments for the Author):

In the manuscript entitled "CRISPR/Cas9 generated MeSWEET10a mutants show reduced susceptibility to cassava bacterial blight and produce viable seed" the authors performed several stable transformations in order to mutate the TALE20 EBE in the promoter of MeSWEET10a. SWEETs are sugar transporters and are known to be induced by Xanthomonas-delivered Type-III effectors (TALEs) to promote virulence. In rice, SWEETs are susceptibility targets, and essential for bacterial colonization of plant tissues. In cassava, however, the contribution of MeSWEET10a induction to virulence (bacterial growth) seems to be rather minor and restricted to water soaking. However, water soaking might lead to an aqueous enviroment in the apoplast, which might benefit bacterial proliferation and spread under field conditions. Therefore, mutating MeSWEET10a promoter in order to turn them insensitive to the corresponding TALE might be a starting point for stacking mutations, leading to Xpm-resistant cassava plants.

Main points:
- The effect of the mutated EBE in the promoter of MeSWEET10a in WT419 seems to be minor, because even without MeSWEET10a induction Xpm is growing similar in the mutant vs the WT cassava plants. One might ask the question whether there is a benefit of MeSWEET10a editing if it affects fertility but not restrict the bacterial growth. However, water soaking differs and could be a starting point for further plant engineering. It would be very interesting to see how these mutant plants behave

under natural condition (in terms of both: fertility and resistance).
- The crossing of mother WT plants with mutant pollen, in my opinion, is not a good assay to analyze the impact on MeSWEET10a promoter mutations on seed development or seed fertility (germination rate). The mother WT plant should carry the meSWEET10a WT allele with the proper expression in corresponding flower tissues. After pollination, seed development is initiated, but here one MeSWEET10a WT allele could be sufficient to ensure normal development. Mutant line #338 possesses two insertions of the T-DNA and therefore could bring in Cas9 and corresponding sgRNAs after pollination, which in turn could mutate the WT allele during seed development. However, this depends on the promoter driving SpCas9 (which is not given in the material and methods, please include this) whether it is expressed during seed development. In addition, even if mutation occurs, the amount of SWEET10a protein produced prior to the edit might be sufficient to allow proper seed development. The better experiment would be to cross the mutant with itself (if possible), then no MeSWEET10a WT copy is present.
- There is also another preprint (Wang et al., 2022, BioRxiv; doi:https://doi.org/10.1101/2022.03.02.482644) with a similar approach in a different Cassava line (60444). Here the authors could show impact on water soaking and bacterial growth after mutation of the TALE20 EBE in MeSWEET10a. The difference could depend on the different cassava line that has been used. However, the authors should at least discuss or compare their findings with the findings of Wang et al., 2022.
- This, unfortunately, left the feeling of lack of novelty. However, depending on the different cassava line and the fact that the other preprint is not published yet. It might be worth publishing. However, there are some critical points I like the authors to address prior to publication (important points)

important points:
- Figure 1 B and E. Line #338 still contains the TALE20 EBE but is not induced by TALE20.
Why is that the case?
Summary of lines:
line TATA EBE UTR CDS Induced Cas9 site T-DNA
copy no.
#269 (108) - - - Δ1-10nt (No ATG) No - homo 2
#338 (108) yes yes yes Δ10-22nt
(frame shift) No - homo 2
#27 (108) - - - +A at 11 nt
(frame shift) no - Homo 2
#30 (108) yes yes Δ11-12nt
(frame shift) no - Homo 4
#41 (249) -/- -/- +T/+C OK no - Het 4
#54 (249) -/- -/- Yes/- OK no Yes
Could block transcription het 3
#69A (249) Yes/yes Yes/yes +T/yes OK yes Yes
Could block transcription het
#69B (108) Yes/Yes Yes/yes Yes/yes Frame shift yes -

I was wondering whether it correlates with the frame shift mutations in the MeSWEET10a coding sequence. But in line #69B MeSWEET10a could be induced although it possesses a frame shift mutation in the MeSWEET10a CDS. The authors should comment on this!
So please discuss why lines #338 and #30 are not induced by TALE20.
In addition, in lines #54 and #69A one Cas9 target (sgR3) is still present and could be targeted or mutated by Cas9. Line #54 carries 3 T-DNA insertions in the genome (line #69A no information). In Line #54 target site for sgRNA3 in one allele should not be stable and can be edited by Cas9.
Phenotyping, particularly in line #338, showed water soaking more similar to the WT plant (supplementary Fig. S7B). Could it be that the RT-PCR data from line #338 are not correct and that there still is induction of TALE20? FigS7C proofs that water soaking is TALE20 dependent.
An important experiment would be a qRT-PCR of these individual lines to have a deeper look into the TALE20-mediated induction of meSWEET10a mutants. Especially for those lines that still carry an intact EBE. (Supplementary fig5S5 is not really convincing)

- Fig4A here I would like to see the expression of MeSWEET10a in flower tissues of the mutant plant #338 in order to compare it to the WT plant.
- Fig4C the anthers look a bit different in the mutant line #338. Microscopy of the pollen might give further insights. If possible provide microscopy pictures of these pollen compared to WT
- The overall germination rate is 41.7 (#338) and 66.7% (WT419). It might be an indication that even under this suboptimal experimental conditions, differences in fertility are already visible. Self-crossing of the mutant line #338 (or another mutant line in which no TALE20 EBE is present anymore) should be performed to address the impact of MeSWEET10a promoter mutations on seed development.

Minor points:
- Supplementary Fig S1: (the donor construct could be visualized in the figure (Ha-length is given in the MM part, but it would be easier for the reader to grasp how the donor has been designed). In addition, the design of the Donor allows Cas9 binding to

target sgRNA1 without cleaving it because the Cas9 DNA binding seed is still intact. This will block the TATA-box and might affect normal MeSWEET10a regulation/expression. I was also wondering why mention the HDR approach at all, but I understand that lines #269 and #338 came from this approach, and needed to be mentioned, because #338 was used for crossing.
- Supplementary Fig. S2 and S3, in both cases labeling could be a bit better. I could identify from which constructs (108,249 or 250) they came from according to the used sgRNAs. However, it would be easier if the construct number (e.g. 108) is also given in the figures (especially fig S3).
- Site 4, on the bottom:
"Line #243 contains a five-base pair (bp) deletion on one allele and a 156 bp deletion on the other allele, both in the promoter region, which may affect TAL effector binding and/or expression. Line #323 is also biallelic, but the mutations are not predicted to affect TAL effector binding or...2
Why mentioning these lines? They do not occur later in the text and confuses the reader. For clarity, you could rather leave them out and only focus on the lines analyzed.
Similar, the approach using the construct 250. There is not a single line analyzed from this transformation. So why mentioning these?

- As mentioned in the discussion, base editors might be an alternative to mutate the EBE only. In addition, one could also use Cas12a with a single crRNA in order to induce medium sized deletions (deletion size peaks at 7 nt).

CRISPR/Cas9 MESWEET10A mutant paper reviewer comments:

Reviewer #1 (Comments for the Author):

Elliott et al. generated MeSWEET10a cassava mutants using CRISPR/Cas9, and claim that these show a reduced susceptibility to cassava bacterial blight. This is an important result but the manuscript has multiple weaknesses, many minor but some more substantial.

1. Abstract: "CBB" is an undeclared abbreviation.

The abbreviation is now defined in the revised abstract.

2. Results: It is unclear how the potential TAL20 effector binding site upstream of the MeSWEET10a start codon was identified. They could explain better why they conclude this is a genuine EBE.

Additional information on how the previous work done to identify the TAL20 EBE site upstream of *MeSWEET10a* was added in the introduction. Additionally, assays used to validate the TAL20 EBE site and *MeSWEET10a* as a susceptibility gene are described.

3. Several combinations of Cas9 guides were used, but there was little explanation of how these guides were selected or what exactly the intended editing outcome is. Were they trying to obtain small indels, a large indel between the guides, a targeted KI or all of these? How were the first pair of guides chosen, followed by additional guides later in the manuscript?

This information is provided in the first paragraph of the Results section and in the materials and methods and has been revised for clarity. In essence, we were attempting all of those possibilities. This work was initiated when gene editing was still quite new, and it wasn't clear which strategy(s) would work best in cassava. Thus, our original construct contained a repair template for potential knock-ins and multiple guides that could either mutate the *MeSWEET10a* gene, the EBE or generate larger deletions. The original theory was that we would recover a bunch of different mutant types and then test to see which worked the best. While we never did see repair template integration, the rest of the strategy worked well in terms of giving us many different types of mutants.

4. Transformation efficiency seems to have been variable, but it was not clearly reported. Can you report a transformation efficiency and how this was calculated?

This is always an interesting point, though challenging to capture accurately with the cassava transformation pipeline. We start with a specific 'settled cell volume' of the FEC, opposed to a known number of cells. To help our readers get a sense of the efficiency, we have added total settled cell volume for each transformed construct. This additional information was added to Table S1.

5. Little effort was made to interpret the mutations' effects on the expression of the gene. It seems very likely that most of the lines will be knock-outs due to the deletion of the start codon, TATA box, or both, but RT-PCR data are required to assess the impact of the mutations. At a minimum, since the authors report that this gene is normally expressed in flowers, they should assess if the mutant alleles they generated are expressed in flowers.

This information was partially captured in Figures 1, 2 and supplemental figure 5, in the original manuscript. RT-PCR was performed on leaf tissue with and without Xpm inoculation to assess the effect of the mutations on ectopic expression of *MeSWEET10a*. However, the reviewer is correct that while we did show expression of *MeSWEET10a* in wildtype flowers, we had not been able to characterize expression in the flowers of the mutant lines. Flowers are very limited and so we have prioritized the available flowers for crosses. While flowers remain limited, we were recently able to collect male and female flowers for *MeSWEET10a* mutant line #338 and performed RT-PCR and compared them to wildtype flower RT-PCR results. This data is presented in supplemental figure 9 and described in the results section. In the case of line #338, transcript was still detected through RT-PCR, though we would predict these RNA molecules to not produce functional proteins, given the frameshift. In the future, we would like to also understand how the lines with other promoter mutations affect endogenous transcription of *MeSWEET10a* in flowers, but these flowers are still very limited and so must be prioritized for self-crosses.

6. It is not clear how they determined the lines' zygosity for the edits. What exactly is meant by "clone-seq"?

We have added additional description of this technique to the Results and Materials and Methods sections. Because of the biallelic nature of most lines, PCR and Sanger sequencing across the target region often produced mixed peaks. Thus, to determine the edits on each allele, gDNA was extracted for each line, the *MeSWEET10a* region was amplified, and the PCR products were cloned into PCR4-TOPO TA vectors. Thus, the two haplotypes were separated into individual plasmids that could be sequenced independently. Multiple colonies were selected per line and screened for those which carried the *MeSWEET10a* PCR product. Plasmid extraction was performed on the colonies, and they were sent for sequencing. The sequences were then compared to the wildtype sequence.

7. They claim to have recovered several T0 plants homozygous for the same NHEJ/MMEJ-induced indels. As these are messy processes, it is surprising that the recovered plants carry two haplotypes with the exact same mutations. Could they be missing alternative haplotypes in the lines they've genotyped? Have they confirmed the lines' inferred zygosity through crossing? I recognize cassava is more difficult to work with than Arabidopsis or tomato, but if they don't know because of technical difficulties, they should make this more clear.

We thank the reviewer for pushing us to investigate this further! Originally, we used clone-sequencing as described in the material and methods section to confirm mutant line genotypes. In many cases, this approach revealed two distinct alleles however, in others, a single mutation type was observed, and this was surprising to us, as well. Due to the timeframe and effort needed to obtain F1 seed for all mutant lines in the field, we are unable to use crosses to separate the alleles. Therefore, and considering the reviewer's comment, we decided to pursue full genome re-sequencing on several lines, as an independent approach to characterizing mutations. In some cases, such as with line #338, this alternative approach allowed us to observe a second mutated allele. In retrospect, we suspect that we were simply unlucky originally, and should have chosen more independent clone-seq colonies to reveal the second allele. Notably, the two alleles in line #338 are similar and so the downstream characterization and interpretation remain unchanged and consistent. This has been updated as described in Figure 1B. However, in other lines, such as #269 and line #30, we were still only able to observe one allele type with the re-sequencing analysis, consistent with our clone-seq data. It is possible that these lines are truly homozygous, though we agree with the reviewer that this seems unlikely. Alternatively, the second allele may have a more complicated re-arrangement or very large deletion that is not resolvable by either method. Based on our RT-PCR data from Xpm-infected leaves for these lines, we conclude that these lines are either homozygous for the observed mutation or have a second mutant allele that prevents TAL20 mediated induction of *MeSWEET10a*. We have updated the manuscript considering this additional characterization and include additional discussion of these points.

8. When they sent samples for Sanger sequencing after digestion, did they also send WT samples? This could have nicely demonstrated that the observed mutations are genuine edits and not just artifacts of the methodology.

Our original mutant line characterization strategy included digestion because we were (overly) optimistic that we might see repair template integration. We agree that sequencing of the wildtype lines would have been a good control. Because we did not see evidence of repair template integration, this assay was abandoned. Importantly, in all subsequent experiments and for all lines included in this manuscript, wildtype lines were included as controls, as described in the materials and methods.

9. When the ability of Xanthomonas to induce the expression of MeSWEET10a was tested i**n Fig. 1E and Fig. 2F,** there was no control for whether the edited lines can still express the gene at all, or if they were knocked-out. Flower tissue from the plants could have provided a nice positive control.

Please see our response to point 5 above. This data is now available in supplemental figure 9.

10. In Fig. 1E and Fig. 2F, you use different Xanthomonas strains. Why?

In Fig. 1E, we chose to use *Xanthomonas euvesicatoria* (Xe) because it has no endogenous TAL effectors and does not cause disease in cassava. Xe modified to carry TAL20 has previously been used as a tool to examine TAL20 specific impacts on susceptibility genes (Cohn and Bart 2014). Using Xe in Fig. 1E served as a proof of concept that the edits in *MeSWEET10a* induction were TAL20 specific without the added complication of eliciting disease in the plant or the presence of additional TAL effectors. After this proof-of-concept experiment, in Fig. 2E we switched to using Xpm, the Xanthomonad that does elicit disease in cassava to show that the edits in *MeSWEET10a* mutant lines prevent TAL20 mediated induction in the native cassava-*Xanthomonas* pathosystem. This has been further clarified in the results.

11. In Fig. 1E and Fig. 2F, you use different housekeeping genes as controls. Why?

In the case of *MeSWEET10a* induction by TAL20, the expression change is so large that both *Actin* and *GTPb* work well. However, during the course of this project, we switched to using the *GTPb* housekeeping gene for other projects in our lab in RT-PCR assays because we found it worked a bit more consistently and provided more reliable results. In the case of small expression changes, we feel that it is best to include two housekeeping genes. But again, for *MeSWEET10* and TAL20, either *Actin* or *GTPb* is sufficient.

12. In Fig. 2 A&B, guides 1, 2, and 3, but not guide 4, are shown. Why? Were no plants sourced from construct 250 genotyped?

Mature lines from construct 250 were collected and genotyped, however lines with mutations of interest for disease phenotyping were selected only from 108 and 249. Genotyping data for all lines including those derived from construct 250 are shown in supplemental figure 4.

13. These results support the hypothesis that edits in these lines block induction of MeSWEET10a by TAL20." Here you interpreted the RT-PCR data as a failure of the pathogen to control the host's metabolism, but the experimental data provided does not bear this out. You may have abolished expression of the MeSWEET10a gene anywhere in the plant.

We believe the reviewer is correctly drawing a distinction between making a knockout mutant versus a promoter mutation that would only block TAL20 induction, not endogenous expression. There are examples in the literature in which gene expression is seen without a TATA box. Therefore, it is possible that the edits to the TATA box/TAL20 EBE and/or 5’UTR may leave gene expression intact. In this revised manuscript we provide additional data on F1 progeny from one such line, line #54, that support this hypothesis. We were able to show that this line can produce viable F1 progeny from both male and female flowers, opposed to line #338 that we believe is female sterile. Longer term, we would like to characterize *MeSWEET10a*

expression in line #54, but these plants are not producing very many flowers currently, as is common in cassava, so we will not be able to provide that data in this manuscript. In the future, we would also like to continue efforts to recover a mutant with edits specific to the EBE with an intact TATA box. We have revised a section in the discussion to highlight these points more clearly and expand upon the priorities for future work.

14. In Figure 3, how were the statistics done?

In Figure 3, statistics were done using an unpaired student's T-Test with unequal variance in microsoft excel. Details on the statistical analysis are provided in the figure legend.

15. When challenged with the Xanthomonas phaseoli pv. manihotis pathogen, the edited lines did not harbour a reduced bacterial population after infiltration. The authors seem to disregard this negative result, and instead accept a reduction in the leaf water-soaked area as a truer sign of reduced disease susceptibility. This is not very convincing and should be more objectively discussed. "We conclude that MeSWEET10a mutant plants have decreased susceptibility to cassava bacterial blight." The current manuscript does not support such a bold claim, since bacterial titres appear unaltered.

Here, the reviewer is asking the interesting and important question, "What does it mean to be 'less susceptible' to a pathogen? And what phenotype or assay is best suited to reveal effects on susceptibility?" The field of plant pathology has a heavy reliance on bacterial growth assays, wherein bacterial populations are monitored over the course of a few hours or days. This method reflects the initial stages of disease, where bacteria colonize the surface of host mesophyll cells and proliferate locally and has the advantage of being relatively consistent and quantitative, compared to visual scoring of disease symptoms that can be subjective and variable. On the other hand, visual scoring of symptoms in the context of a full leaf or plant is more closely related to what we care about in agriculture. Further, bacterial growth in a single spot may be ideal for leaf spot pathogens, but for systemic pathogens, such as Xpm, bacteria quickly enter the vasculature and spread throughout the plant, so bacterial growth at the site of inoculation only captures that early phase of the disease cycle. Our goal is to make cassava lines that will show decreased susceptibility to CBB in the field. Will bacterial growth at the site of inoculation or disease symptoms prove to be more reflective of disease in the field? We do not yet know. However, especially considering the details of the Xpm disease cycle, we argue that bacterial growth at the site of inoculation is not obviously more important or relevant than disease symptoms, in terms of understanding field level susceptibility.

We do know that TAL20 plays a major role in the water-soaked disease symptom phenotype and a lesser role in bacterial growth. As shown in figure 3 and supplemental figure 7, a difference between Xpm WT and Xpm△TAL20 was not consistently observed in edited lines or wildtype cassava plants. There are a number of technical reasons for why a difference might

not be captured and it is possible that alternative strategies for bacterial growth assays would reveal a larger difference in Xpm growth. However, we have invested significant effort into optimizing these assays, testing different time points and conditions and the result is always the same (perhaps a slight impact on growth, but not consistent). In other pathosystems water-soaking has been uncoupled from bacterial growth as a virulence mechanism for pathogens. Therefore, for characterizing these lines, we feel strongly that the disease symptom is the more appropriate assay. In the discussion section, we elaborate on potential technical and biological reasons for why we do not observe a difference in bacterial populations and why water-soaked lesions alone are a reasonable measure of disease severity.

17. "line #338 had been established in a field site outside Hilo, Hawaii along with WT419 plants. WT419 female and male flower buds were collected for RNA extraction and RT-PCR. Expression of MeSWEET10a was confirmed for both flower types" It is surprising that the authors tested the expression of MeSWEET10a in the WT line but did not test in the mutant.

Please see our response to point 5 above. This data is now available in supplemental figure 9.

18. Discussion: "The ideal MeSWEET10a mutant line, for CBB resistance, would contain edits at the TAL20 EBE site while maintaining an intact TATA box and gene coding sequence for native plant function." This sentence needs to be moved to the early Results/late Introduction.

We agree and have added this context in multiple places throughout the revised manuscript.

19. "It is also possible that other TAL20 carrying Xpm strains may have effectors with redundant functions and therefore may not exhibit reduced virulence after infection in MeSWEET10a mutants." Is Xpm ΔTAL20 less virulent?

Please see our response to point 15 for a lengthy discussion on how lab assays translate (or not) to field phenotypes. But the short answer is yes. We observe reduced water-soaked lesions from XpmΔTAL20 infected WT plants compared to Xpm WT. This data has been previously published (Cohn and Bart 2014 MPMI, Elliott 2022 Plant Methods, Veley et al 2023 Nature Comms) and is also shown in this manuscript (Figure 3).

Reviewer #2 (Comments for the Author):

In the manuscript entitled "CRISPR/Cas9 generated MeSWEET10a mutants show reduced susceptibility to cassava bacterial blight and produce viable seed" the authors performed several stable transformations in order to mutate the TALE20 EBE in the promoter of MeSWEET10a. SWEETs are sugar transporters and are known to be induced by Xanthomonas-delivered Type-III effectors (TALEs) to promote virulence. In rice, SWEETs are susceptibility targets, and essential for bacterial colonization of plant tissues. In cassava, however, the

contribution of MeSWEET10a induction to virulence (bacterial growth) seems to be rather minor and restricted to water soaking. **However, water soaking might lead to an aqueous enviroment in the apoplast, which might benefit bacterial proliferation and spread under field conditions. Therefore, mutating MeSWEET10a promoter in order to turn them insensitive to the corresponding TALE might be a starting point for stacking mutations, leading to Xpm-resistant cassava plants.**

Main points:
1. The effect of the mutated EBE in the promoter of MeSWEET10a in WT419 seems to be minor, because even without MeSWEET10a induction Xpm is growing similar in the mutant vs the WT cassava plants. One might ask the question whether there is a benefit of MeSWEET10a editing if it affects fertility but not restrict the bacterial growth. However, water soaking differs and could be a starting point for further plant engineering. It would be very interesting to see how these mutant plants behave under natural condition (in terms of both: fertility and resistance).

Thank you for your comments. We agree that editing *MeSWEET10a* is a good starting point for engineering CBB disease resistance and also that it is important to understand the effects on fertility and field level resistance. For the latter, this is the longer-term goal of this project. We will first identify F1 progeny with the transgene segregated away and then move forward to field trials in target countries, such as Nigeria, Uganda or Kenya. This will be a 2-3 year process, best case scenario, so is outside the scope of the current manuscript. However, for fertility, we were able to collect additional important data for line #338 and #54, that are now included in this manuscript. This new data demonstrates the importance of mutating the EBE while leaving the *MeSWEET10a* coding sequence intact.

2. The crossing of mother WT plants with mutant pollen, in my opinion, is not a good assay to analyze the impact on MeSWEET10a promoter mutations on seed development or seed fertility (germination rate). The mother WT plant should carry the meSWEET10a WT allele with the proper expression in corresponding flower tissues. After pollination, seed development is initiated, but here one MeSWEET10a WT allele could be sufficient to ensure normal development. Mutant line #338 possesses two insertions of the T-DNA and therefore could bring in Cas9 and corresponding sgRNAs after pollination, which in turn could mutate the WT allele during seed development. However, this **depends on the promoter driving SpCas9 (which is not given in the material and methods, please include this)** whether it is expressed during seed development. In addition, even if mutation occurs, the amount of SWEET10a protein produced prior to the edit might be sufficient to allow proper seed development. The better experiment would be to cross the mutant with itself (if possible), then no MeSWEET10a WT copy is present.

Cas9 was expressed by a 35s promoter which is now clarified in the materials and methods. Thank you for pointing out this omission. We also appreciate the reviewer pointing out that it is possible for an active CRISPR/Cas9 to continue editing a WT allele introduced through crossing, and we were able to investigate this possibility a bit further. We have obtained additional seed from several different crossing types and include their characterization in this revised manuscript. Additional crossing attempts were performed for line #338 and we obtained the first seed from line #54 self-crosses and crosses to wildtype. This additional work revealed several important findings. First, it is now clear that while line #338 readily produces viable F1 progeny from male flowers, the female flowers are not reproductively competent. Thus, we conclude that expression of *MeSWEET10a* is essential in female flowers. We still do not see an obvious developmental defect in line #338 female flowers, but the additional crossing data is convincing. As for whether the active Cas9 might cause further edits in the wildtype allele introduced through crosses, we now have some evidence that this is possible. Specifically, we molecularly characterized several F1 progeny from line #338 or line #54 x WT crosses and see evidence of the original allele on one haplotype and a novel second allele on the other haplotype. For the reasons pointed out by the reviewer, we think this is to be expected.

Importantly, our goal with this project is to generate new cassava varieties that could be distributed to farmers that show increased tolerance to CBB. While the female flower defect would not prohibit clonal propagation of lines, it would hinder further breeding efforts. Thus, we focused our efforts on generating F1 progeny from line #54, which contains promoter mutations but an intact *MeSWEET10a* coding sequencing. Excitingly, we were able to recover a handful of viable F1 progeny from line #54 female flowers. To date, these plants are not producing very many flowers (they were planted in the field relatively recently) and so we have needed to prioritize every available flower for crossing experiments opposed to other molecular analyses. However, we hypothesize that the promoter mutations in line #54 do not block endogenous expression of *MeSWEET10a* in female flowers. All of this new data is included in this revised manuscript with accompanying discussion.

3. There is also another preprint (Wang et al., 2022, BioRxiv; doi:https://doi.org/10.1101/2022.03.02.482644) with a similar approach in a different Cassava line (60444). Here the authors could show impact on water soaking and bacterial growth after mutation of the TALE20 EBE in MeSWEET10a. The difference could depend on the different cassava line that has been used. However, the authors should at least discuss or compare their findings with the findings of Wang et al., 2022. This, unfortunately, left the feeling of lack of novelty. However, depending on the different cassava line and the fact that the other preprint is not published yet.

We saw this pre-print when it posted and went back and forth on whether we should cite this related work. As the reviewer points out, the manuscript has not yet appeared in a peer reviewed format. If the reviewer and editor insist, we will cite the bioRxiv paper, but our preference would be not to do so. As is clear from our own peer review process, peer review often reveals aspects of the work that need to be further considered and sometimes the peer review process changes interpretations, etc. Thus, it feels premature to cite the pre-print in a peer reviewed manuscript. As of the time of submitting these revisions, the above-mentioned pre-print has not yet been published.

It might be worth publishing. However, there are some critical points I like the authors to address prior to publication (important points) important points:

4. Figure 1 B and E. Line #338 still contains the TALE20 EBE but is not induced by TALE20. Why is that the case? I was wondering whether it correlates with the frame shift mutations in the MeSWEET10a coding sequence. But in line #69B MeSWEET10a could be induced although it possesses a frame shift mutation in the MeSWEET10a CDS. The authors should comment on this! So please discuss why lines #338 and #30 are not induced by TALE20.

While Sanger sequencing did show a potential 1bp deletion after the TSS for line #69B, this edit was not well resolved by sanger-seq alone. This was a rapid assay to identify lines to further characterize and because we saw evidence of TAL20 mediated induction based on RT-PCR results for Xpm infected #69B detached leaves (Supplemental figure 6), we did not pursue this line further. As for the lines that do show conclusive evidence of frameshift mutations but with the TATA box and TAL20 EBE intact, for example line #338, originally, we hypothesized that the frameshift mutations caused nonsense-mediated mRNA decay (NMD), and that was why the transcript was not observed after Xpm inoculation. However, our newly acquired data from line #338 flowers has left us scratching our heads. In flowers, we do see transcript produced from line #338. These transcripts would not produce a functional protein, given the frameshift mutations, but the data shows that at least in flower tissue, transcript is still produced. So why don't we see transcript in leaves after Xpm inoculation? The possibilities we have discussed include different regulatory mechanisms driving expression in leaves (with TAL20) versus endogenous expression in flowers. Alternatively, perhaps the magnitude of induced expression in leaves after TAL20 binding triggers NMD and the relatively lower expression level in flowers does not trigger this process. While we do not currently fully understand this result, we have included an expanded discussion of the topic, as recommended by the reviewer.

5. In addition, in lines #54 and #69A one Cas9 target (sgR3) is still present and could be targeted or mutated by Cas9. Line #54 carries 3 T-DNA insertions in the genome (line #69A no

information). In Line #54 target site for sgRNA3 in one allele should not be stable and can be edited by Cas9.

We agree with the reviewer's interpretation of sgR3 in line #54 and have added additional consideration to this point in the discussion of the manuscript. Because 69A is a silent mutant not of interest for crossing in the field, we did not include the line for WGS and T-DNA insertion analysis, though here too we agree with the idea that the gRNAs may continue to be active, until the binding site has been disrupted.

6. Phenotyping, particularly in line #338, showed water soaking more similar to the WT plant (supplementary Fig. S7B). Could it be that the RT-PCR data from line #338 are not correct and that there still is induction of TALE20? FigS7C proofs that water soaking is TALE20 dependent. è An important experiment would be a qRT-PCR of these individual lines to have a deeper look into the TALE20-mediated induction of meSWEET10a mutants. Especially for those lines that still carry an intact EBE. (Supplementary fig5S5 is not really convincing).

Considering all the data for line #338, we are confident that there is a reduction in water-soaked disease symptoms, and that this is TAL20 dependent. However, we also agree with the reviewer that the phenotype is less extreme than some of the other lines. We think the most likely explanation for this is somaclonal variation coming out of tissue culture, which is common in cassava. However, it could also reflect the differences in the mutant alleles themselves. We have added additional text to the discussion on potential variation among the mutant genotypes and, importantly, emphasizing the desired objective of obtaining lines with the EBE mutated while maintaining the coding sequence. It is OK that Supplementary figure 5 is not as convincing because this was used as an initial screen. The most interesting lines were then further characterized and confirmed, and the corresponding data is provided in the main figures.

7. Fig4A here I would like to see the expression of MeSWEET10a in flower tissues of the mutant plant #338 in order to compare it to the WT plant.

We agree and have addressed this point in the responses above.

8. Fig4C the anthers look a bit different in the mutant line #338. Microscopy of the pollen might give further insights. If possible provide microscopy pictures of these pollen compared to WT

Unfortunately, we were unable to obtain flowers for microscopy from this line. While we did not notice any major visible differences in flowers used for macro imaging, we do agree that a future line of investigation would be to compare mutant and WT flowers by microscopy as mentioned in the discussion. Our current data suggest that it might be most porductive to focus this work on the female reproductive organs. These experiments are not impossible but

are also not trivial since these lines do not flower easily in greenhouses or growth chambers and even in the field, flower production is limited. Additionally, comparing flowers that are synchronized in development will be needed. In our study, we were limited by what was flowering in the field and selected flowers that were as close in developmental stage as possible and we acknowledge these limitations.

9. The overall germination rate is 41.7 (#338) and 66.7% (WT419). It might be an indication that even under this suboptimal experimental conditions, differences in fertility are already visible. Self-crossing of the mutant line #338 (or another mutant line in which no TALE20 EBE is present anymore) should be performed to address the impact of MeSWEET10a promoter mutations on seed development.

We are thankful that both reviewers and editor pushed us to lean into the fertility characterization through additional crosses. This took a significant amount of time and effort but was worth it since we now have a more accurate understanding of the role of *MeSWEET10a* in flowers and the types of edits that we need to obtain to achieve our ultimate goal. Our results have been added to the manuscript and described in the responses above.

Minor points:

10. Supplementary Fig S1: (the donor construct could be visualized in the figure (Ha-length is given in the MM part, but it would be easier for the reader to grasp how the donor has been designed). **In addition, the design of the Donor allows Cas9 binding to target sgRNA1 without cleaving it because the Cas9 DNA binding seed is still intact. This will block the TATA-box and might affect normal MeSWEET10a regulation/expression.** I was also wondering why mention the HDR approach at all, but I understand that lines #269 and #338 came from this approach, and needed to be mentioned, because #338 was used for crossing.

To clarify how the donor was designed, we have updated Supplemental Fig. 1B to include the full repair template sequence. The maps for all constructs are also provided in supplemental materials. The reviewer's comment about the DNA binding seed still being intact and potentially interfering with the transcriptional machinery is interesting, though further experimental work would be needed to investigate.

13. Supplementary Fig. S2 and S3, in both cases labeling could be a bit better. I could identify from which constructs (108,249 or 250) they came from according to the used sgRNAs. However, it would be easier if the construct number (e.g. 108) is also given in the figures (especially fig S3).

The construct number has been added for each line to improve clarity (Supp Fig 4).

14. Line #243 contains a five-base pair (bp) deletion on one allele and a 156 bp deletion on the

other allele, both in the promoter region, which may affect TAL effector binding and/or expression. Line #323 is also biallelic, but the mutations are not predicted to affect TAL effector binding. Why mentioning these lines? They do not occur later in the text and confuses the reader. For clarity, you could rather leave them out and only focus on the lines analyzed. Similar, the approach using the construct 250. There is not a single line analyzed from this transformation. So why mentioning these?

We made these lines and characterized their genotypes with sanger-sequencing. Although we did not characterize these lines further, there may still be a small amount of value to including this data in terms of better understanding the types of mutations recovered in cassava. To avoid confusing the reader, we removed the description of line #243 and #323 from the text of the results section and refer to “Additional lines that were not characterized further are included in the supplemental materials”. This is also the case for lines generated from construct 250.

15. - As mentioned in the discussion, base editors might be an alternative to mutate the EBE only. In addition, one could also use Cas12a with a single crRNA in order to induce medium sized deletions (deletion size peaks at 7 nt).

This point has been added to the discussion.

22-Feb-2024

Dr. Rebecca Bart
Donald Danforth Plant Science Center
Bart Lab
St. Louis, Missouri

RE: CRISPR/Cas9 generated MeSWEET10a mutants show reduced susceptibility to cassava bacterial blight and produce viable seed

Dear Dr. Rebecca Bart:

We are pleased to accept your manuscript for publication in the Plant Physiology. This acceptance is contingent on revision based on the comments of our reviewers. In particular, please consider the following:

Please highlight all changes and include a detailed annotation to changes to the text, with line numbers, and noting your responses to the comments.

To submit your revised manuscript, click:

Link Not Available

If you cannot return the revised manuscript within 8 weeks of receipt, please let us know. Otherwise, we will assume that you have elected not to revise the manuscript and are withdrawing it.

Thank you for allowing us to review your work. We look forward to hearing from you soon.

Sincerely,

Board of Editors, Plant Physiology

-- Reviewer comments:
Reviewer #1 (Comments for the Author):

The Bart lab have made many reasonable responses to the reviewer's comments

I see the main merit of this paper as illustrating how hard it can be to achieve useful outcomes via gene editing, despite the public discourse that seems to suggest it is the solution to any agricultural problem that might be addressed by crop improvement.

I would highlight 4 specific but related shortcomings

1- It is clear that resistance has not been achieved; at best, the edited lines show a slight reduction in symptoms

2- most of the CrispR-induced mutations are likely to be knock-outs. It's not clear that the authors have achieved the goal of mutating the target site for the Xanthomonas TAL effector without having any effect on either the reading frame or the endogene wild type expression patterns and protein function.

3 They report that gene is no longer inducible by the pathogen, but in the examples tested eg 338 it looks like the gene is dead via deletion into post translation initiation sequence

The authors should be far more explicit about what proportion of their events are likely complete loss of function. I would include a TATA deletion as likely loss of function unless the authors provide compelling evidence that that allele still shows wild type levels and patterns of expression.

The authors report > 100 crossing attempts with line 338 and line 54. Surely they could have made RNA from ~ 5 of those flowers to test SWEET gene expression

I do acknowledge and sympathise with the fact that cassava is a great deal harder to work with than eg rice where there are nice reports of editing promoters for bacterial blight resistance. I think this report would do a greater service to the community by

being very upfront about how much more difficult cassava is to improve by gene editing

Reviewer #2 (Comments for the Author):

I would like to thank the authors for taking our comments into consideration. The manuscript is much better now.

Just three minor things I spotted (Point 2 and 3 are up to the authors).

Minor:

1. Introduction:

"The DNA binding domain directs the TAL effector to predictable DNA sequences, called effector binding elements (EBEs) [(Cernadas et al., 2014), (Moscou and Bogdanove, 2009), and (Boch and Bonas, 2010)]. In many cases, TAL effector binding causes upregulation of downstream susceptibility genes."

To be fair, please also cite Boch et al., 2009 beside Moscou and Bogdanove 2009. These papers, describing the TALE code, have been published back to back.

2. Figure S6
The labeling is shifted a bit. Would look better, if the labels are in line with the lanes in the gel.

3. Discussion
"In F1 progeny in which a wildtype allele has been crossed in, we may also expect to see new alleles arise, if the Cas9 machinery continues to express."

For clarity I suggest to write:
"In F1 progeny in which a wildtype allele has been crossed in, we may also expect to see new alleles arise, if the Cas9 machinery continues to express and enable Cas9-mediated target cleavage."

Reviewer #1 (Comments for the Author):

The Bart lab have made many reasonable responses to the reviewer's comments

I see the main merit of this paper as illustrating how hard it can be to achieve useful outcomes via gene editing, despite the public discourse that seems to suggest it is the solution to any agricultural problem that might be addressed by crop improvement.

I would highlight 4 specific but related shortcomings

1- It is clear that resistance has not been achieved; at best, the edited lines show a slight reduction in symptoms
We agree and have been careful to not overstate the strength of the effect, throughout the manuscript. Additional consideration of this topic is given in the discussion.

2- most of the CrispR-induced mutations are likely to be knock-outs. It's not clear that the authors have achieved the goal of mutating the target site for the Xanthomonas TAL effector without having any effect on either the reading frame or the endogene wild type expression patterns and protein function.
The data suggest that the TATA box may be dispensable for MeSWEET10a gene expression, since line #27 was able to produce viable seed. We have added additional focus to this topic in the discussion and we agree that we have not yet achieved the ideal mutant.

3 They report that gene is no longer inducible by the pathogen, but in the examples tested eg 338 it looks like the gene is dead via deletion into post translation initiation sequence

The authors should be far more explicit about what proportion of their events are likely complete loss of function. I would include a TATA deletion as likely loss of function unless the authors provide compelling evidence that that allele still shows wild type levels and patterns of expression.
We have captured this data in Table 1 and hope it will help clarify exactly what we have produced through this work.

The authors report > 100 crossing attempts with line 338 and line 54. Surely they could have made RNA from ~ 5 of those flowers to test SWEET gene expression

I do acknowledge and sympathise with the fact that cassava is a great deal harder to work with than eg rice where there are nice reports of editing promoters for bacterial blight resistance. I think this report would do a greater service to the community by being very upfront about how much more difficult cassava is to improve by gene editing
We appreciate this feedback and have added additional text to the discussion, as requested. We look forward to further characterizing MeSWEET10a expression in flowers in the future. These lines have now stopped flowering and it is hard to know when they might start again. The joys of working with cassava!

Reviewer #2 (Comments for the Author):

I would like to thank the authors for taking our comments into consideration. The manuscript is much better now.
Thank you for the kind words. We have made all the suggested revisions.

Just three minor things I spotted (Point 2 and 3 are up to the authors).

Minor:

1. Introduction:

"The DNA binding domain directs the TAL effector to predictable DNA sequences, called effector binding elements (EBEs) [(Cernadas et al., 2014), (Moscou and Bogdanove, 2009), and (Boch and Bonas, 2010)]. In many cases, TAL effector binding causes upregulation of downstream susceptibility genes."

To be fair, please also cite Boch et al., 2009 beside Moscou and Bogdanove 2009. These papers, describing the TALE code, have been published back to back.

2. Figure S6
The labeling is shifted a bit. Would look better, if the labels are in line with the lanes in the gel.

3. Discussion
"In F1 progeny in which a wildtype allele has been crossed in, we may also expect to see new alleles arise, if the Cas9 machinery continues to express."

For clarity I suggest to write:
"In F1 progeny in which a wildtype allele has been crossed in, we may also expect to see new alleles arise, if the Cas9 machinery continues to express and enable Cas9-mediated target cleavage."

02-Apr-2024

Dr. Rebecca Bart
Donald Danforth Plant Science Center
Bart Lab
St. Louis, Missouri

MSID: PP2023-RA-00918R2
MS TITLE: CRISPR/Cas9 generated MeSWEET10a mutants show reduced susceptibility to cassava bacterial blight and produce viable seed

Dear Dr. Rebecca Bart:

I am pleased to inform you that your revised manuscript "CRISPR/Cas9 generated MeSWEET10a mutants show reduced susceptibility to cassava bacterial blight and produce viable seed" has been accepted for publication in Plant Physiology. Your manuscript will be checked for consistency with journal formatting and image standards by one of our Science Editors, who will contact you shortly. They may have a number of queries and suggestions for you. Please keep in mind that they work to increase the online visibility of your article and its attraction for readers from the widest of backgrounds. You will have the opportunity to review any revisions, and I hope you will work constructively with them to get the most out of your research. You will also have the opportunity to review all changes when you receive the author proofs for your article.

Once validated for publication, your article will be sent to a Science Editor for a final review of your manuscript and source files. After your final files are uploaded, your article will appear in the next available issue and the online Preview version of your article will be posted after your signed Copyright and Checklist forms and final source files are received.

Please be sure to include the following in your manuscript file:
Harvard style citations
List of all author contributions and funding information at the beginning of manuscript after the titles and author list.
List all supplemental materials associated with the article, with titles, at the end of the ms, before the references and acknowledgments section.
Complete Figure Legends for all figures appearing in the article listed before the Literature Cited.

Thank you and congratulations on your Plant Physiology paper!

Sincerely,
Board of Editors Plant Physiology

=====
FIRST AUTHOR PROFILES
=====

We recognize the hard work that goes into being First Author of a Plant Physiology paper by publishing a "First Author Feature" for each First Author alongside your paper.

If the first author(s) would like to submit information for a feature on Facebook and Twitter, fill out the form by clicking on the link below:

Plant Physiology First Author Feature Form: https://forms.office.com/r/ZBCGZfhm8F
If you cannot access the form through the link above, please contact Rachel at rbelsky@aspb.org.

To upload an image of yourself to accompany your profile, please put your photo in the correct journal folder at the link below. You must label your image: Author First Name Last Name Article Title.

First Author Profile Image Dropbox: https://www.dropbox.com/scl/fo/xz029p7qjr2jml2rp2kmq/h?dl=0&rlkey=6fkxcaqmxx35ap152b2osy1fz

=====

IMPORTANT REMINDER: PEER REVIEW REPORTS
=====

If you opted to publish a peer review report along with your article during the original submission process, it will be prepared by the editorial staff and publicly posted with your manuscript, inside the zip file that contains any other supplemental material. As a reminder, the peer review report is a public record of all comments from editors and reviewers, as well as your prior responses, as you received them in the decision letters for each draft of your manuscript. If you agreed to publish this report and have changed your mind, or are not sure if you selected this option, please contact the editorial office as soon as possible before signing the license agreement from our publisher.

-- Reviewer comments:
